# Heterotelechelic homopolymers mimicking high *χ* – ultralow *N* block copolymers with sub-2 nm domain size†

**DOI:** 10.1039/d2sc00720g

**Published:** 2022-03-14

**Authors:** E. Hancox, M. J. Derry, M. J. Greenall, S. Huband, L. Al-Shok, J. S. Town, P. D. Topham, D. M. Haddleton

**Affiliations:** Department of Chemistry, University of Warwick Coventry CV4 7AL UK d.m.haddleton@warwick.ac.uk; Aston Institute of Materials Research, Aston University Birmingham B4 7ET UK p.d.topham@aston.ac.uk; School of Mathematics and Physics, University of Lincoln Brayford Pool Lincoln LN6 7TS UK; Department of Physics, University of Warwick Coventry CV4 7AL UK

## Abstract

Three fluorinated, hydrophobic initiators have been utilised for the synthesis of low molecular mass fluoro-poly(acrylic acid) heterotelechelic homopolymers to mimic high chi (*χ*)–low *N* diblock copolymers with ultrafine domains of sub-2 nm length scale. Polymers were obtained by a simple photoinduced copper(ii)-mediated reversible-deactivation radical polymerisation (Cu-RDRP) affording low molecular mass (<3 kDa) and low dispersity (*Đ* = 1.04–1.21) homopolymers. Heating/cooling ramps were performed on bulk samples (*ca.* 250 μm thick) to obtain thermodynamically stable nanomorpologies of lamellar (LAM) or hexagonally packed cylinders (HEX), as deduced by small-angle X-ray scattering (SAXS). Construction of the experimental phase diagram alongside a detailed theoretical model demonstrated typical rod–coil block copolymer phase behaviour for these fluoro-poly(acrylic acid) homopolymers, where the fluorinated initiator-derived segment acts as a rod and the poly(acrylic acid) as a coil. This work reveals that these telechelic homopolymers mimic high *χ*-ultralow *N* diblock copolymers and enables reproducible targeting of nanomorphologies with incredibly small, tunable domain size.

## Abstract 2

Three fluorinated, hydrophobic initiators have been used for the synthesis of low molecular mass fluoro-poly(acrylic acid) heterotelechelic homopolymers to mimic high chi (*χ*)–low *N* diblock copolymers with ultrafine domains of sub-2 nm length scale.

## Graphical abstract

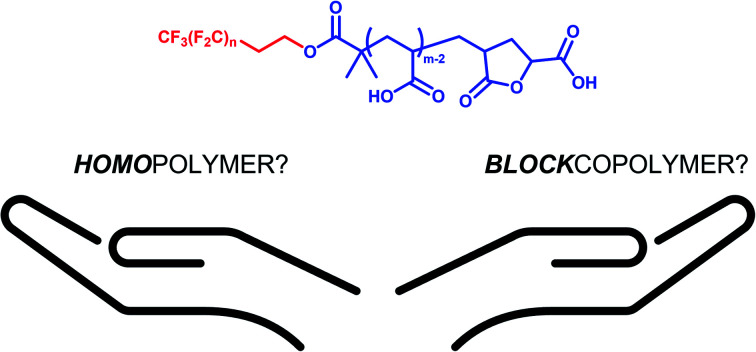

## Introduction

Diblock copolymers have been widely used for many different applications including drug delivery,^1–3^ superhydrophobic materials,^4–6^ nanolithography,^7–9^ photonics,^10^ actuators,^11–14^ water filtration^15^ and thermoplastic elastomers.^16–18^ Block copolymers find use in bulk self-assembly applications due to the vast amount of design possibilities. Many ordered nanostructures (lamellae, double gyroid, hexagonally packed cylinders, body centred cubic)^19–21^ can be accessed by tuning the antagonistic interaction properties between blocks. This has piqued interest in the microelectronics industry, which constantly strives for (long range, defect-free) smaller domain spacings.^22^ Block copolymer microphase separation is a bottom-up approach that overcomes current issues^23^ and allows for tailorable chemical functionalities and mechanical properties. The thermodynamic driving force for self-assembly is given by the incompatibility between disparate segments, often achieved through large differences in polarity or hydrophobicity.^24,25^ This interaction parameter (Flory–Huggins interaction parameter, *χ*) must exceed a critical value (χ*) to induce microphase separation in typical coil–coil block copolymers,^26^ where χ* = 10.5/*N* and *N* = *N*_A_ + *N*_B_ (*N* is the total number of monomer units and *N*_A_ and *N*_B_ are the number of monomer units in each block).^27^ The movement towards using block copolymers to obtain the smallest domain spacings has established a class of “high *χ* – low *N*” block copolymers in which *N* is minimised to reduce domain size, whilst *χ* is maximised to retain a block interaction parameter capable of microphase separation despite the entropic penalty associated with demixing. Balancing these factors to maintain a sufficiently high value of *χN* to enable self-assembly has led to sub-5 nm (ref. 28–30) and sub-3 nm (ref. 31 and 32) feature sizes. Low *N* block copolymers that maintain *χN* ≥ 10.5 often contain a very hydrophobic part that can incorporate siliconised, fluorinated or styrenic groups (within the backbone^33–35^ or side chain^30,36–38^) combined with a hydrophilic/ionic block.^39^ Morphology can also be manipulated by introducing secondary interactions^40^ or branching points^41^ to control molecular packing in solution.^42^ Bottlebrush copolymers have also been shown to influence phase behaviour, and provide a route to densely packed architectures.^43^ Thermodynamic equilibrium in microphase separation is typically achieved by thermal or solvent annealing, but selective solvent vapour annealing can also be used to access kinetically-trapped morphologies.^44^ The ultimate goal is to obtain a defect-free, single grain morphology (long range order), highlighting the importance of the annealing process and understanding the factors that influence microphase separation. Long range order is obtained by removing grain boundaries, formed by slow coarsening kinetics that can prevail over a thermodynamic driving force. A block copolymer's ability to produce a defect-free structure is given by the coarseness of the template and the degree of coupling between the template and block copolymer.^45^

Copper-mediated reversible-deactivation radical polymerisation (RDRP) (often called atom-transfer radical polymerisation, ATRP) can achieve very high control over polymer molecular mass and dispersity,^46,47^ and is therefore an ideal candidate to synthesise low molecular mass polymers. However, it is often difficult to achieve very low molecular mass polymers using RDRP techniques. Typically, the rate of termination (*k*_t_) is highest in the initial stages of polymerisation and modelling suggests an exponential increase in *k*_t_ from DP 100 to DP 1.^48^ This implies that the synthesis of a polymer with DP < 10 with low dispersity (*Đ* < 1.2) is essentially impossible if standard free radical principles are obeyed, yet control over dispersity is required to achieve well-ordered nanoscale morphologies in the bulk.^49^ The Haddleton group has previously shown that this is achievable with a photoinduced reduction of copper(ii), which led to the smallest domain size so far reported, to the best of our knowledge (for a homopolymer),^50^ as well as achieving full monomer conversion in <2 minutes,^51^ which confronts the dead chain fraction equation that assumes all chains must terminate.^52^ Rapid and controlled free radical polymerisation is almost a misnomer as rapid living radical polymerisation requires high radical concentration, however, a high concentration of radicals should lead to increased termination *via* normal radical–radical termination events. Ballard and Asua offered an elegant explanation for these “improbable” scenarios and, contrary to the predictions from free radical polymerisation, bimolecular termination is avoided when rapid radical deactivation is present. Thus, using a diffusion factor explains situations that would otherwise be considered impossible.^53^ A second explanation put forward by Szymanski is that there is an interaction between the propagating chain and the transition metal complex giving rise to two type of.^54^ The complexed or caged radical forms a second type of radical which have different rate constants of termination. This second explanation is considered plausible and in contrast to the widely held view of a free radical process^55,56^ Using this phenomenon, one is able to exploit the photoinduced reduction of copper(ii) to obtain very low molecular mass polymers with low dispersity.^57–59^

Herein, we report a straightforward synthetic route to yield poly(acrylic acid) heterotelechelic homopolymers that exhibit exquisite ordered bulk nanomorphologies at sub-2 nm length scales for homopolymers. The materials could also be considered as diblock copolymers with a short PTFE block and a longer PAA block but we chose not to use this interpretation as there is no mass distribution in the first part of the molecule thus not conforming to the definition of a polymer which must always contain a mass distribution. Fluorinated initiators are employed as discrete, fluorinated segments to yield poly(acrylic acid) homopolymers with ultralow *N*. We previously demonstrated the use of the F_13_ initiator to prepare a small sub-set of poly(acrylic acid) samples *via* the facile deprotection of Cu-RDRP generated poly(*tert*-butyl acrylate). In the present work, we explore the self-assembly behaviour and map out the phase diagram for these bulk nanomorphologies, enabling control of morphology with tuneable domain size. To the best of our knowledge, this previous work was the first to achieve sub-2 nm domain sizes *via* polymer microphase separation for homopolymers.^50^ Herein, synthesis *via* Cu-RDRP yielded three series of homopolymers with *M*_*n*,NMR_ <3 kDa and *Đ* = 1.04–1.12, showcasing the ability to generate well-defined ultralow *N* heterotelechelic homopolymers. These polymers (F_*n*_-PAA_*m*_) were thermally annealed, and small-angle X-ray scattering (SAXS) revealed lamellar and hexagonally packed cylindrical morphologies with 1.7–3.9 nm domain sizes, depending on their compositional position in the phase diagram. Transitions of both order-to-order and order-to-disorder were observed and a mathematical investigation of the molecular mass dependency of the domain spacing was conducted, based on modelling the fluorinated segment as a rod. Crucially, we reveal that “high *χ* – low *N*” block copolymers are not needed to obtain highly segregated polymers with sub-2 nm domains. Instead, new heterotelechelic homopolymers with ultralow *N* are shown to mimic rod–coil block copolymers, offering an accessible and straightforward route to highly segregated polymers with controllable morphology and domain sizes down to 1.7 nm.

## Results and discussion

In the present work, a short poly(tetrafluoroethylene)-like fluorocarbon chain is used in the synthesis of an alkyl halide initiator, to act as a hydrophobic moiety. The two methylene units decouple the electronic effects of the electron withdrawing CF_2_ groups from the oxygen atom, leading to reactivity resembling an alkyl alcohol, such as ethanol. This ensures a low, discrete molecular mass for the fluorinated segment to be maintained, providing an overall decrease in the final polymer's potential molar mass dispersity since only one “polymeric” segment is formed by polymerisation.

**Scheme 1:**
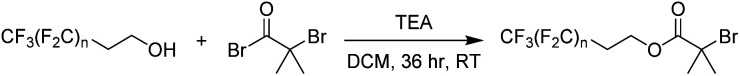
Synthesis of fluorocarbon initiators. *n* = 5 (F_13_), 7 (F_17_), 9 (F_21_).

Three different alkyl halide initiators were synthesised by esterification of α-bromoisobutyryl bromide with perfluorocarbon alcohols containing 13, 17 or 21 fluorine atoms (Scheme 1). These initiators are referred to as F_13_, F_17_ and F_21_ herein (^1^H, ^13^C, ^19^F NMR shown in Fig. S1–S5†). Of experimental note, DCM was found to be a suitable solvent for the synthesis of F_13_ and F_17_. However, chloroform was required for the synthesis of F_21_ as the increase in halogen affinity increased solubility of 1*H*,1*H*,2*H*,2*H*-perfluoro-1-dodecanol. Poly(acrylic acid) (PAA) was chosen as the polar, hydrophilic segment as it is easily synthesised *via* deprotection of poly(*tert*-butyl acrylate) (P*t*BA), and we have previously shown that well-defined PAA with molecular mass <5 kDa can be synthesised *via* this route.^50^ The interaction parameter, *χ*, is clearly large between PAA and the hydrophobic PTFE-like segment, since strong segregation of these materials is observed.

**Scheme 2:**
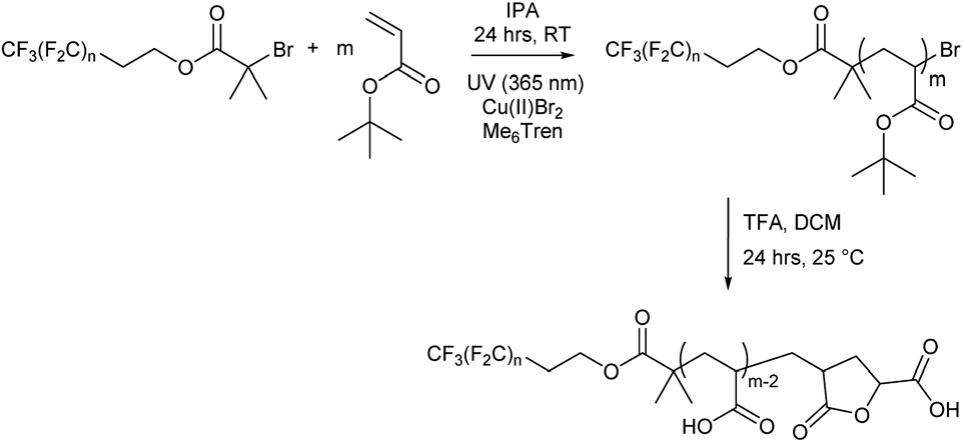
Synthesis of fluoro-acrylic acid polymers (F_*n*_-PAA_*m*_).

Each fluorocarbon initiator was used to synthesise P*t*BA (F_*n*_-P*t*BA_*m*_) allowing for comparisons of both segment lengths independently, covering a large area of the phase space when mapping out their nanoscale behaviour. Photoinduced copper(ii)-reversible deactivation radical polymerisation (Cu(ii)-RDRP) was used for the synthesis of P*t*BA (Scheme 2), which can be deprotected to give the desired PAA. The photoinduced synthesis allows for excellent control over molecular mass, even at very low DP, with little observable termination and near-perfect end group fidelity, despite the rapid polymerisation, and thus very low dispersity for all products. Polymerisations were performed in isopropanol as solvent, resulting in shorter reaction times than with DMSO, which has previously been shown to be effective since P*t*BA becomes insoluble in DMSO above a certain molecular mass, and we were mindful to avoid polymerisation-induced self-assembly (PISA) or precipitation.

**Fig. 1:**
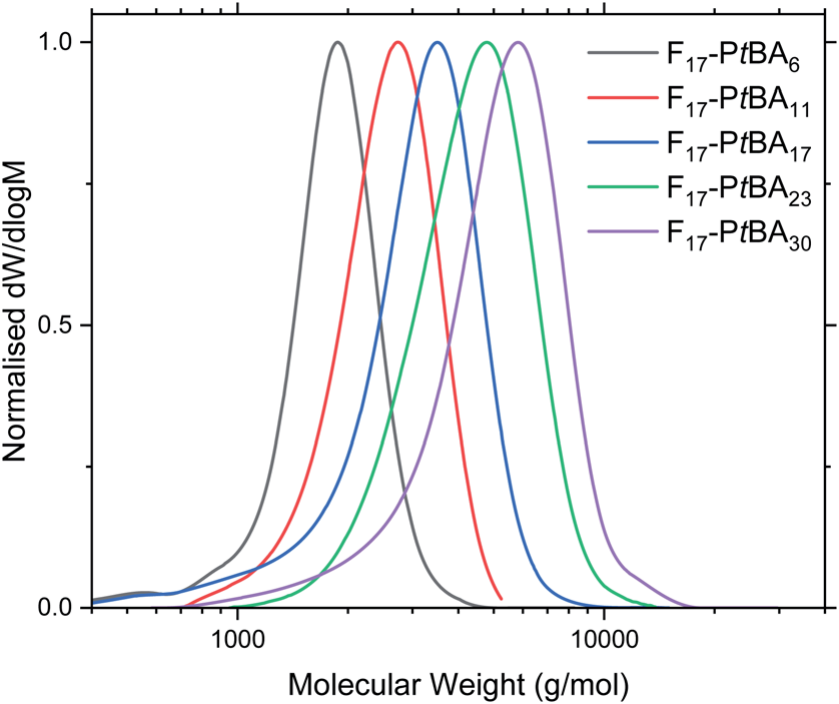
GPC traces of F_17_-P*t*BA_*m*_ in THF eluent. See Fig. S12–S13† for F_13_-P*t*BA_*m*_ and F_21_-P*t*BA_*m*_.

F_*n*_-P*t*BA_*m*_ polymers were obtained in high yield (P*t*BA monomer conversion ≥ 95% in all cases), as confirmed by ^1^H NMR spectroscopy. The number-average molecular mass (*M*_*n*_) and degree of polymerisation for F_*n*_-P*t*BA_*m*_ were also determined using ^1^H NMR (Table 1 and Fig. S6–S8†). This method was not used to determine the DP of F_*n*_-PAA_*m*_ (Fig. S9–S11†) as it was believed that the amphiphilic nature of the polymers could alter the real peak intensities in the ^1^H NMR spectrum due to insufficient solubility and possible solution aggregation.^60–62^ Additionally, the *t*-butyl peak is more reliable for integration than polymer backbone peaks or the acidic proton in poly(acrylic acid). For similar reasons, THF eluent was used for GPC analysis for F_*n*_-P*t*BA_*m*_ polymers. The GPC traces show narrow, unimodal molar mass distributions and low molecular mass (Table 1 and Fig. 1), indicating good control throughout the polymerisation, including the early stages. Due to these complications we believe the most reliable measure of molecular weight (and thus DP) comes from the ^1^H NMR of the F_*n*_-P*t*BA_*m*_ polymers in CDCl_3_.

**Table 1:** F_*n*_-PAA_*m*_ polymer characteristics

Polymer^a^ F_*n*_-PAA_*m*_	Molecular mass^a^ (*M*_*n*_, g mol^−1^) (NMR)	Molecular mass^b^ (*M*_*n*_, g mol^−1^) (GPC)	Dispersity^b^	Volume fraction (*f*_F_)	*N* c	Nano-morphology^d^	d*^d^ (nm)
F_13_-PAA_4_	800	1400	1.08	0.42	9	LAM	1.7
F_13_-PAA_5_	870	1450	1.06	0.37	10	LAM	1.9
F_13_-PAA_6_	945	1500	1.11	0.34	11	LAM	2.0
F_13_-PAA_9_	1160	1900	1.11	0.26	14	HEX	2.4
F_13_-PAA_11_	1310	1950	1.10	0.23	16	HEX	2.6
F_13_-PAA_12_	1380	2000	1.17	0.21	17	DIS	(2.2)
F_13_-PAA_15_	1590	2650	1.08	0.18	20	DIS	(2.3)
F_13_-PAA_17_	1740	2700	1.14	0.17	22	DIS	(2.5)
F_13_-PAA_18_	1810	3400	1.10	0.16	23	DIS	(2.6)
F_13_-PAA_22_	2100	3300	1.14	0.13	27	DIS	(2.7)
F_13_-PAA_25_	2510	4300	1.13	0.12	30	DIS	(2.9)
F_13_-PAA_27_	2460	3900	1.17	0.11	32	DIS	(2.9)

F_17_-PAA_6_	1040	1600	1.13	0.39	12	LAM	2.1
F_17_-PAA_11_	1410	2400	1.11	0.27	17	HEX	3.0
F_17_-PAA_17_	1840	2600	1.24	0.20	23	HEX	3.1
F_17_-PAA_23_	2270	4000	1.15	0.16	29	HEX	3.4
F_17_-PAA_30_	2770	4600	1.21	0.13	36	HEX	3.7

F_21_-PAA_5_	1070	2000	1.04	0.47	12	LAM	2.0
F_21_-PAA_10_	1430	2200	1.13	0.33	17	LAM	2.8
F_21_-PAA_16_	1870	2800	1.14	0.24	23	LAM	2.9
F_21_-PAA_20_	2150	3900	1.16	0.20	27	HEX	3.8
F_21_-PAA_24_	2440	4100	1.15	0.18	31	HEX	3.9

a Degree of polymerisation and number-average molecular mass determined by ^1^H NMR of F_*n*_-P*t*BA_*m*_ polymers in CDCl_3_.

b THF GPC data against poly(methyl methacrylate) standards.

c Total polymer degree of polymerisation.

d Determined by SAXS measurements of single data acquisition samples. DIS = disordered, LAM = lamellar, HEX = hexagonally packed cylinders, d* = domain size (half-pitch), calculation for HEX given in Fig. S37. Domain sizes, bracketed, for disordered morphologies are calculated from the principal peak for completeness, but long-range order is not observed in these samples due to the lack of higher order peaks.

All F_*n*_-P*t*BA_*m*_ polymers were subsequently deprotected to give amphiphilic F_*n*_-PAA_*m*_ heterotelechelic homopolymers as white solids. Polymer structure was confirmed by ^1^H NMR and MALDI-TOF-MS, where all spectra of the latter showed a repeat unit of 72 g mol^−1^, consistent with acrylic acid repeat units. Multiple distributions were present in the MALDI-TOF-MS spectrum and can all be accounted for (Fig. S14–S17†). The dominant molecular mass distribution showed loss of both a H and a Br atom indicating that the polymer has a terminal vinyl group from elimination of HBr following polymerisation, or more likely, cyclisation occurring during the deprotection step/or MS sample preparation resulting in a lactone moiety.^63^ This likely increases rigidity and at low molecular weights, may have significant influence on microphase separation. However, hydrogen bonding between lactone rings and acrylic acid is thought to counter any negative influences induced by rigidity.^64^ Molecular mass also showed the fluorocarbon moiety was not removed during deprotection. Full experimental details are given in the ESI.

**Fig. 2:**
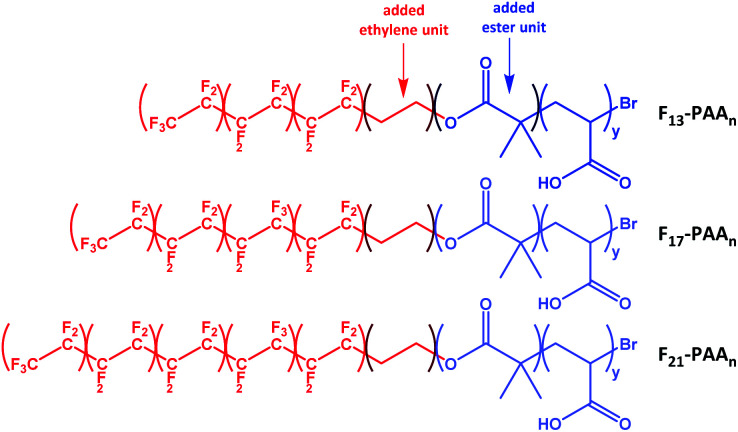
Compartmentalisation of the polymers used to calculate the volume fractions of each segment (fluorinated segment in red and PAA segment in blue).

*N* and volume fractions (*f*_F_ and *f*_PAA_) were calculated, where *N* is the sum of units in each segment (*N* = *N*_F_ + *N*_PAA_), and are given in Table 1 (calculations given in Fig. S34†). To calculate the volume fraction of each segment, the fluorinated segment was treated as tetrafluroethylene (TFE) repeat units. This compartmentalisation is shown in Fig. 2, in which F_13_, F_17_ and F_21_ comprises 3, 4 and 5 TFE units, respectively. The C_2_H_4_ spacer was approximated to have the same volume as one TFE repeat unit, therefore increasing *N*_F_ to 4, 5, and 6, respectively. It is important to note that in the volume fraction calculation, the ester and ethylene linking moieties were treated as belonging to the segment that they most closely resemble in terms of chemical functionality. In short, the ester linkage was included as part of the hydrophilic PAA block (and attributed to the same volume as an acrylic acid unit, so that *N*_PAA_ is given by the polymer DP calculated by ^1^H NMR, plus one for the ester group) and the apolar ethylene unit as part of the fluorinated block. For simplicity, the end-group cyclisation and rearrangement depicted in Scheme 2 has not been used in this calculation.

**Fig. 3:**
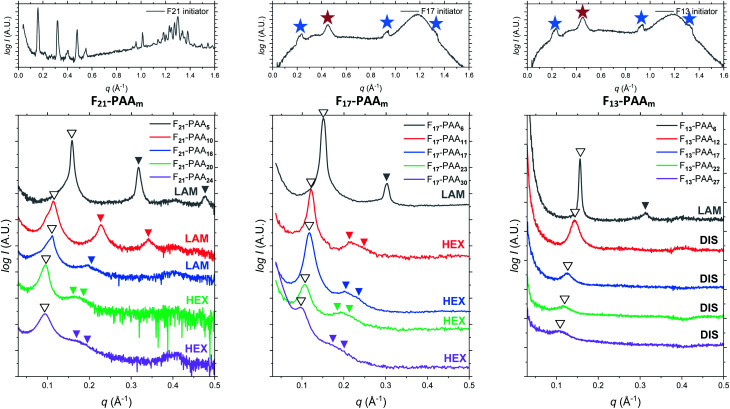
Single data SAXS measurements for thermally annealed F_*n*_-PAA_*m*_ heterotelechelic homopolymers using F_21_, F_17_ and F_13_ initiators (left to right). Open arrows show principal peak (q*) position. Closed arrows indicate theoretical peak positions of the labelled morphology (LAM = lamellar, HEX = hexagonal cylinders, DIS = disordered). SAXS profiles of F_21_, F_17_ and F_13_ initiators (PAA DP = 0) given above. Blue stars indicate artefacts from between detectors, red stars indicate Kapton.

Differential scanning calorimetry (DSC) was used to determine the glass transition temperature (*T*_g_) for each polymer (Fig. S18–S22†). Measurements were performed for all F_13_-, F_17_- and F_21_-PAA_*m*_ polymers in addition to a hydrogenated (non-fluorinated) control with no amphiphilic character (named EBiB-PAA_*m*_). EBiB-PAA_*m*_ polymers were synthesised following the same procedures as for the F_*n*_-PAA_*m*_ polymers. The trend in *T*_g_ is in accordance with the Flory–Fox equation, where increasing molecular mass gives a logarithmic increase in *T*_g_. This trend reaches a plateau in *T*_g_ at approximately 110 °C for all fluorinated telechelic homopolymers as the DP exceeds 30. It is important to know this upper limit in *T*_g_ when selecting the temperature at which to perform thermal annealing experiments, as this sets a minimum temperature for polymer reorganisation within the bulk state. All polymers were annealed to induce bulk microphase separation and achieve thermodynamic equilibrium (full details for annealing are given in the ESI†). Two types of SAXS experiment were conducted: (i) single data acquisition after thermal annealing and (ii) time-resolved measurements during thermal annealing, referred to as ‘single data’ and ‘time-resolved data’, respectively. Thermal annealing was achieved by heating the bulk polymer to 120 °C (>*T*_g_ for all polymers) for 24 hours then allowing them to slowly cool to room temperature. The thermally annealed polymers were then analysed using small-angle X-ray scattering (SAXS) to investigate microphase separation (Fig. 3). The highest intensity peak at lowest scattering vector, *q*, is the principal peak (q*) and is used to determine the interplane spacing from which the domain spacing (often referred to as “full-pitch”) is calculated within the polymer morphology. For lamellae (LAM), this is given by *d* = 2π/q* and for hexagonally packed cylinders (HEX), *d* = (2π/q*)/sin(60), where *d* is the domain spacing. Considering all F_*n*_-PAA_*m*_ data, the average domain size, denoted as d* (where d* = *d*/2, and gives the upper limit for the minimum single domain size assuming both domains are equal, often referred to as “half-pitch”), range from 1.7 nm to 3.9 nm. As the fluorocarbon length is constant, domain size increases as the PAA chain length increases. Similarly, increasing the fluorocarbon length increases domain size, which is visible when considering the trend of all three sets (Fig. S23†), as expected. The arrows on the SAXS profiles show the theoretical position of higher order peaks for the labelled morphology, where the first three expected peaks for LAM are at q*, 2q* and 3q*, and those for HEX are at q*, √3q* and 2q*, to enable self-assembled morphologies to be assigned.^65^ For both the F_21_-PAA_*m*_ and F_17_-PAA_*m*_ polymer sets, the morphology changes from LAM to HEX as the PAA chain length increases, which is well-known for typical block copolymers due to a decrease in the volume fraction of one of the blocks; in this case, the fluorocarbon segment, *f*_F_, in our homopolymers. The F_13_-PAA_*m*_ polymer set also transitions from LAM (*N* = 11) to HEX (*N* = 14, 16) to DIS (*N* = 17) when data from our previous preliminary work is included. Additionally, the SAXS profiles for the F_21_, F_17_, and F_13_ initiators have been placed above their respective polymer sets. F_21_ is a crystalline solid at room temperature and LAM peaks from the crystallites are visible in the scattering profile. F_17_ and F_13_ initiators are both liquids at room temperature and were measured in a capillary tube using Kapton (peak observed at *q* ∼ 0.45 Å^−1^ in the scattering patterns), as well as artefacts that are a result of integrating from 2D to 1D due to the gaps in the detector. There is no other indication of any order, other than a slight difference in intensity of the amorphous peak at ∼1.2 Å^−1^, a profile for the blank capillary can be found in Fig. S24.† The initiators were not thermally annealed prior to the measurements, acting only as a control for the polymers. A summary of all domain sizes and morphologies determined from single data acquired after thermal annealing are given in Table 1.

The short and crystalline nature of the F_21_ initiator justifies the assumption that the *N*-dependence of the domain spacings in the F_21_-PAA_*m*_ polymers can be reasonably described by a model in which the F segment is treated as a rod and the PAA segment as a coil. Further support for this approach is provided by the fact that the Kuhn length of PTFE is 2.3 nm,^66^ longer than the F segment itself and also around 4–5 times longer than the Kuhn lengths found in simulations^67^ and experiments^68^ for PAA. In the limit where the incompatibility between the two segments is high, as we propose for the current polymers, Müller and Schick^69^ developed a simple model for block copolymers, based purely on competition between the interfacial free energy and the stretching cost of the coil block, that provides formulae that we have used to fit the domain spacing data for the different morphologies. These formulae have one adjustable parameter that is common to all morphologies, and we take the approach of fitting the domain spacings of the F_21_-PAA_*m*_ polymers in the lamellar phase, where the justification for using the rod–coil model is strongest, then using the value of the parameter found here to predict the domain spacings for all other samples.

**Fig. 4:**
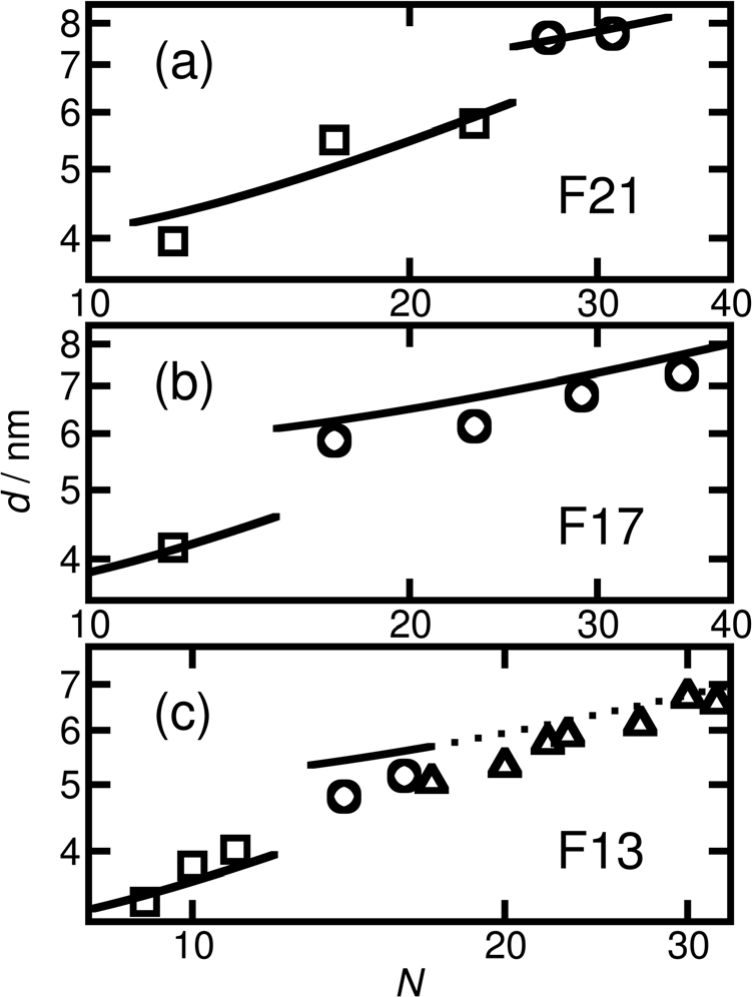
Domain spacing (full pitch) *versus N* for the thermally annealed (a) F_21_-, (b) F_17_- and (c) F_13_-PAA_*m*_ samples. The experimental values are shown by squares for LAM, circles for HEX and triangles when the morphology is uncertain/disordered. For the purposes of comparison with the prediction of the model for the domain spacing in the HEX phase, the inter-plane spacings for the uncertain morphologies have been converted to centre-to-centre distances by multiplication by 2/√3. The left-hand line in (a) shows a fit found using a model of strongly segregated rod–coil polymers. The other solid lines show the predictions of this model, using the value of the fitting parameter from the first fit, for the domain spacing in the other samples. The prediction for the spacings in (c) whose morphology is uncertain is shown with a dotted line.

In the model of Müller and Schick,^69^ the domain spacing in the lamellar phase is given by eqn (1):1
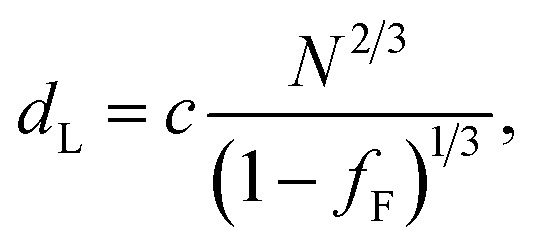
where *c* is the adjustable parameter referred to above. As in our earlier work,^50^ the similarity of the repeat unit volumes of PAA and PTFE means that *N* can be used as given in Table 1 and there is no need to normalise it to the repeat unit volume of one of the segments. The result of fitting the F_21_-PAA_*m*_ LAM full-pitch domain spacings with this formula is shown in Fig. 4(a). Given that only one free parameter is used, the agreement is good.

The prediction of the model^69^ for the domain spacing in the HEX phase is shown in eqn (2):2
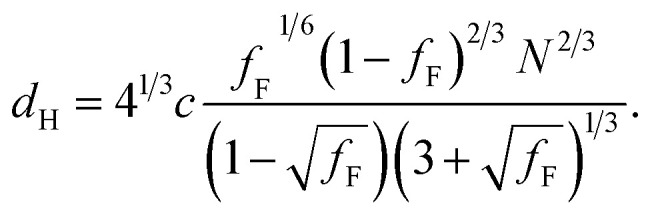


Using this formula with the value for *c* found by fitting the F_21_-PAA_*m*_ LAM data gives an accurate prediction for the domain spacings in the F_21_-PAA_*m*_ HEX phase, also shown in Fig. 4(a).

Since *c* does not depend on *N* or *f*_F_, the value determined from the F_21_-PAA_*m*_ LAM fit can continue to be used to predict the domain spacings for the F_17_-PAA_*m*_ polymers. In this case (Fig. 4(b)), an accurate result is found for the value of *d* for the sample in the lamellar phase. Although the numerical values of the predictions of eqn (2) for the domain spacings in the F_17_-PAA_*m*_ HEX phase are slightly too high, the slope of the experimental data on a log–log plot is reproduced well. The prediction of the rod–coil model for this slope is better than those of the standard strong segregation^70^ (*d* ∝ *N*^2/3^) and weak segregation^71^ (*d* ∝ *N*^1/2^) formulas derived for coil–coil polymers, which give significantly steeper slopes than seen here, where approximately, *d* ∝ *N*^0.3^.

Furthermore, eqn (1) and (2) can be used with the same value of *c* to predict the domain spacings for the F_13_-PAA_*m*_ polymers. The results of these calculations are shown in Fig. 4(c). Although the higher-order peaks in the SAXS data are too weak for the final seven F_13_-PAA_*m*_ data points to be unambiguously identified as HEX, there is evidence from TEM^50^ that the F_13_-PAA_18_ (*N* = 23) sample forms a weakly ordered array of cylinders and it is reasonable to compare the domain spacings in these samples with the predictions of the model for the HEX phase. These inter-plane spacings have therefore been converted to centre-to-centre distances by multiplication by 2/√3 before being plotted in Fig. 4(c). Good agreement is then found between these values and the predictions of the model for the HEX phase, with the shallow slope (*d* ∝ *N*^0.4^) of the final nine points reproduced well.

Given the simplicity of the rod–coil model and the fact that only one fitting parameter is used, the agreement obtained between the model and the experimental data across the different morphologies is good. It is important to note that the model^69^ does not account for the location of the LAM-HEX transition in the current data and predicts it to lie at a much higher value of *f*_F_ (0.765) than seen here, possibly due to the fact that anisotropic interactions^69^ and the “shortness” of the molecules^72^ are not taken into account. Nevertheless, given the good quality of the fits and the clear physical motivation for using a rod–coil model, we believe that a more detailed theory of this form (*e.g.*, one including a Maier–Saupe treatment of the anisotropic interactions^72^) would be a promising future line of investigation.

**Fig. 5:**
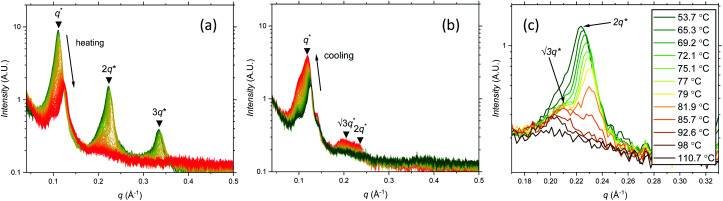
Time-resolved SAXS measurements of F_21_-PAA_10_. Colour scale from green to red shows the (a) heating cycle (30 °C to 150 °C at 0.5 °C min^−1^) and (b) cooling cycle (150 °C to 30 °C at 0.5 °C min^−1^) (c) expansion of the second peak (heating cycle, Fig. 5(a)).

Further SAXS studies were conducted to understand the phase behaviour of these polymers by investigating the order–disorder transitions (ODT) and any order–order transitions (OOT). Time-resolved SAXS measurements were conducted on bulk heterotelechelic homopolymers that were prepared in the same manner as for the single data SAXS measurements to further probe the phase behaviour of these polymers during thermal annealing. The bulk samples were heated (and subsequently cooled) at 0.5 °C min^−1^ (the slowest ramp rate achievable for the experimental set-up) from 30 °C to 150 °C to 30 °C, while collecting data every 1 minute (*i.e.* every 0.5 °C). This ramp rate was selected to provide the best chance for the system to attain thermal equilibrium. The heating and cooling cycles from time-resolved SAXS of F_21_-PAA_10_ and video representations were created to assist visualising the time-resolved SAXS data (Movies S1–S5†), Fig. 5. On heating, the intensity of the peaks decreased, and higher order peaks disappeared completely, indicating a loss in ordered morphology at the order-disorder transition temperature (ODT). This is expected, as *χ* has an inverse relationship with temperature (*χ* = *A* + *B*/*T*, where *A* and *B* are constants for a given pair of chemical entities). There is also an observed shift in the position of the principal peak (q*) to larger *q* values, meaning a decrease in domain spacing during the heating process, which returns to its original position on cooling. The intensity and higher order peaks also return during cooling, indicating good thermoreversibility. Almost all of the samples return to their original morphology upon cooling, with the exception of F_21_-PAA_10_ and F_21_-PAA_16_ (Table 2). Notably, the higher order peaks of these two samples change from relative positions of *q* = q*, 2q*, 3q* to *q* = q*, √3q*, 2q*, demonstrating a change in morphology from lamellar (LAM) to hexagonally-packed cylinders (HEX), which indicates that HEX is the thermodynamically stable morphology for these polymers.

**Table 2:** Characteristics of F_*n*_-PAA_*m*_ polymers from time-resolved SAXS measurements

Polymer F_*n*_-PAA_*m*_	*T*_ODT, midpoint_ (°C)	*T*_g_ a (°C)	Nano-morphology *before* thermal annealing	Nano-morphology *after* thermal annealing
F_17_-PAA_6_	—	52.1	LAM^b^	LAM^b^
F_17_-PAA_11_	—	78.8	HEX^b^	DIS/HEX^b^
F_17_-PAA_17_	—	100.0	HEX^b^	HEX^b^
F_17_-PAA_23_	—	108.4	HEX^b^	HEX^b^
F_17_-PAA_30_	—	111.6	DIS^b^	DIS^b^

F_21_-PAA_5_	94.0^c^	77.9	LAM	LAM
F_21_-PAA_10_	87.4	86.9	LAM	HEX
F_21_-PAA_16_	102.9	99.5	LAM	HEX
F_21_-PAA_20_	105.8	109.6	HEX	HEX
F_21_-PAA_24_	—	110.0	HEX	HEX

a Taken from DSC data.

b Temperature measurements were recorded with heating/cooling rates of 5 °C min^−1^.

c See Fig. 6.

Fig. 5(c) shows a magnification of the region around the second peak (heating cycle, Fig. 5(a)) from the time-resolved SAXS data of F_21_-PAA_10_. The formation of a new peak at √3q* appears, indicating an order–order transition (OOT) and provides a degree of confidence in the phase boundaries presented in the phase diagram (see later). Such thermally-induced transitions can be rationalised when inspecting the experimental phase diagram, since an increase in temperature corresponds to moving downwards in the phase diagram, as *χ* = *A* + *B*/*T*. F_17_-PAA_*m*_ polymers were also annealed in a similar manner, however the inherent reduction in *N* leads to weaker peak intensities making it difficult to pinpoint the *T*_ODT_. Consequently, time-resolved SAXS measurements were not performed during annealing for the F_13_-PAA_*m*_ polymers.

**Fig. 6:**
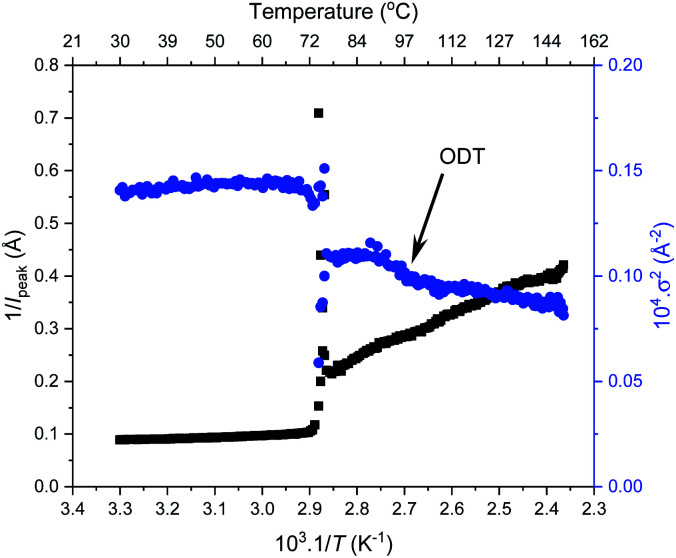
Peak intensity (*I*_peak_) and full width half maximum (*σ*) of the principal peak from thermal SAXS heating cycle *vs.* 1/temperature for F_21_-PAA_5_. Transition temperatures = 73.6 °C and 94.0 °C (*T*_ODT_).

To investigate the order–order and order-disorder transitions, the time-resolved data were plotted as the inverse peak intensity of the principal peak at q* (1/*I*_peak_) *versus* reciprocal temperature (1/*T*). Fig. 6 shows this for F_21_-PAA_5_ alongside the inverse of full width half maximum squared (1/*σ*^2^) of the principal peak, plots for the remaining F_21_-PAA_*m*_ polymers are given in Fig. S25–S33.† The sharp decrease in peak intensity signifies the temperature at which a transition occurs, which is corroborated by a sharp increase in 1/*σ*^2^ at the same temperature. To determine the transition temperatures, three linear trendlines were fitted to the plots: before, after and during the sharp change in intensity. The midpoint of the two intersections was taken as the transition temperature (the fitted data are given in Fig. S25–S33†). All polymer transition temperatures and morphology changes are given in Table 2. *T*_OOT_ or *T*_ODT_ values were only calculated for four polymers, as those with higher PAA content are less ordered and the change in q* intensity is insufficient to fit three distinguishable straight lines, due the lack of discernible phase transition, see Fig. 6.

**Fig. 7:**
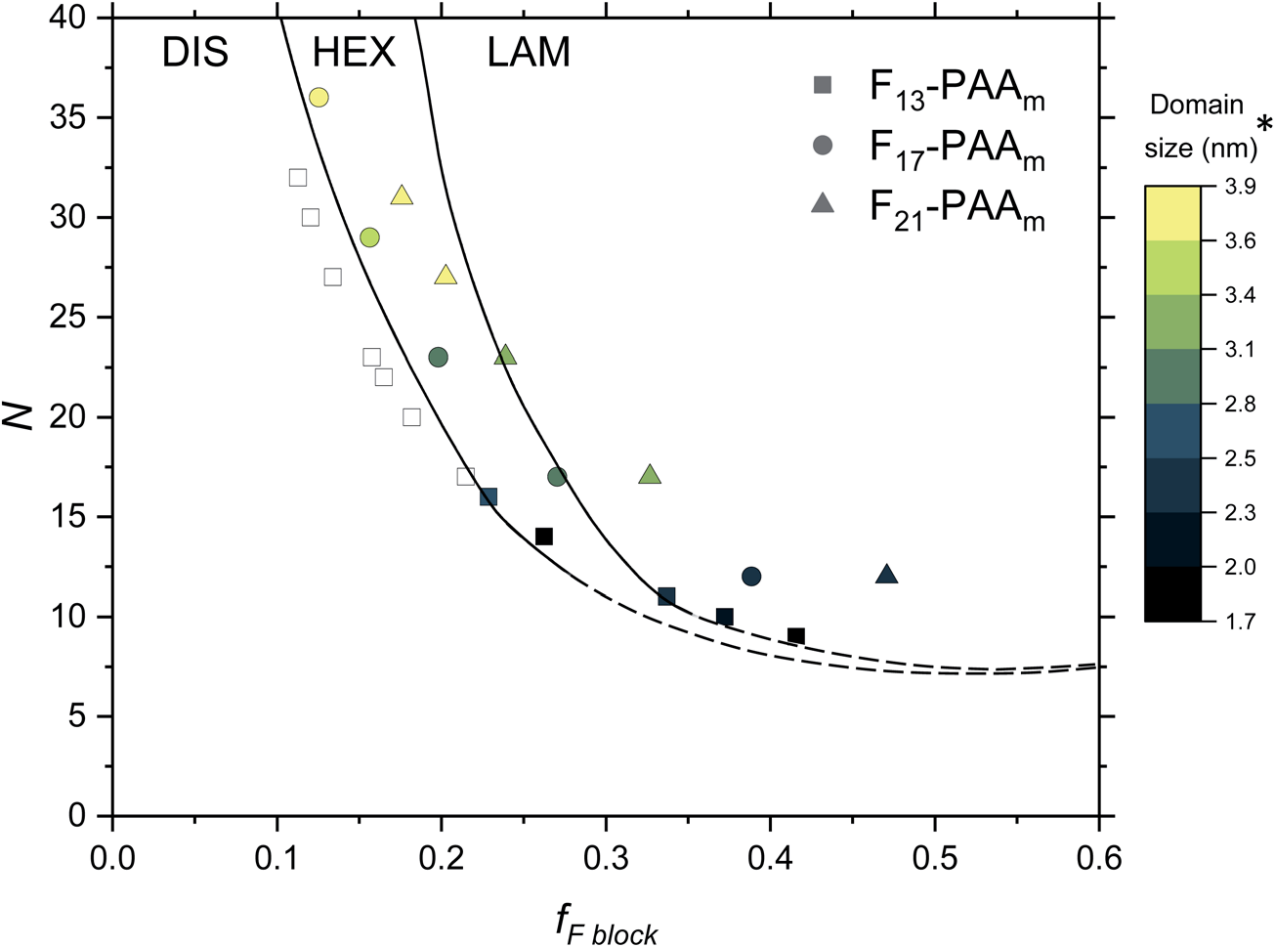
Phase diagram for the F_*n*_-PAA_*m*_ telechelic homopolymers, including data obtained in previous research.^50^ Morphologies taken from single data SAXS acquisition after thermal annealing. DIS = disordered, HEX = hexagonally packed cylinders, LAM = lamellae. Note that the phase boundaries are not absolute and are only a guide to the eye. *The domain size (d*) is estimated as the half-pitch, which provides the upper limit for the minimum single domain size assuming that both domains are equal.

Finally, an experimental phase diagram was constructed from the SAXS data (Fig. 7), including F_13_-PAA_*m*_ data from our previous study.^50^ Although typical phase diagrams use *χN* for the *y*-axis, on the basis of *χ* being a constant value at a given temperature, the shape of our phase diagram (*N versus f*_F_) can be directly compared to those in the literature. The morphologies obtained from a single data acquisition after thermal annealing were used to construct the phase diagram where three clear regions have been identified; DIS, HEX and LAM. Phase boundaries were placed as a guide to the eye and are not absolute, however, a solid line has been used to show more certain boundary areas, where data points either side show order. Remarkably, our experimental phase diagram for these telechelic homopolymers closely resembles that of the theoretical rod–coil block copolymer phase diagram with no liquid crystal alignment.^73^ The colour bar in Fig. 7 indicates domain size (d*) for heterotelechelic homopolymers that phase separated into ordered morphologies. Disordered polymer samples are shown as open squares. F_13_-PAA_*m*_ polymers (where *N* = 14 & 16) are weakly ordered (HEX according to SAXS data), as such both of these data points lie very close to the phase boundary. Construction of this phase diagram enables reproducible targeting of particular nanomorphologies with given domain sizes, a particularly powerful tool in molecular engineering modern materials. The domain sizes typically decrease on decreasing *N* and increasing *f*_F_ across the range of telechelic homopolymers in this study, attributed to the shorter molecular lengths associated with low *N* and tighter packing of rod segments. The morphology changes observed during time-resolved measurements at a slower cooling cycle give an approximate indication of the phase boundaries. The OOT observed for F_21_-PAA_10_ is represented by a shift downwards in the phase diagram due to the decrease in *χ* on heating (Fig. S35†). Slower cooling rates in time-resolved measurements allow the polymer to self-assemble into a more thermodynamically stable state compared to rapid cooling, where samples could become kinetically trapped in a less preferential state. This work shows a strong resemblance to the theoretical phase diagram for a rod–coil block copolymer (Fig. S36†),^73^ highlighting the remarkable similarity in phase behaviour between our telechelic homopolymers and rod–coil block copolymers. Our work paves the way for a new generation of modern materials that can access extremely small domain sizes, exploiting high *χ*-low *N* principles in simple homopolymers.

## Conclusions

A library of heterotelechelic poly(acrylic acid) homopolymers have been shown to strongly mimic the self-assembly behaviour of rod–coil block copolymers in the bulk. Fluorinated initiators were utilised as a hydrophobic moiety in the preparation of homopolymers that exhibit sub-2 nm domains, offering an alternative approach to traditional high *χ*–ultralow *N* block copolymers. Synthesised by Cu-RDRP, highly amphiphilic homopolymers were obtained with high conversion (>95%) and low dispersity (*Đ* <1.21) *via* deprotection of a *tert*-butyl protecting group.

Single data SAXS measurements showed equilibrium morphologies of LAM, HEX and DIS, allowing the phase diagram to be constructed. Theoretical studies using rod–coil and coil–coil models were performed to provide further confidence in our interpretation of the experimental data. A rod–coil model in the strong segregation regime was shown to have good agreement for all of the polymers in this study.

This work demonstrates a possible solution to the ever-present desire for smaller domains in the microelectronics industry. We show that appropriate polymer end groups are a powerful asset for achieving strong microphase separation and offer a procedure in which the smallest domain sizes and morphologies can be targeted.

## Data availability

Raw data including DSC, GPC, MALDI TOF MS, NMR and SAXS (including movies) is available to download at https://wrap.warwick.ac.uk/163791/.

## Author contributions

E. H. synthesized the initiators and polymers and carried out NMR, GPC, DSC, SAXS data collection, M. J. D. assisted with the direction, interpretation and the analysis of the SAXS experiments and data analysis, M. J. H. performed fitting, modelling and assisted in analysing and interpretation of the SAXS data, S. B. collected SAXS data with E. H. and assisted in the interpretation of the data, L. A.-S. and J. S. T. collected and assisted in the interpretation of the MALDI-ToF-MS data, P. D. T. directed the SAXS experimentation and analysis of the results D. M. H. conceived, supervised and led the collaborative work. E. H., P. T. and D. M. H. wrote the manuscript with the support and contribution of all authors.

## Conflicts of interest

There are no conflicts to declare.

## Supplementary Material

SC-013-D2SC00720G-s001

SC-013-D2SC00720G-s002

SC-013-D2SC00720G-s003

SC-013-D2SC00720G-s004

SC-013-D2SC00720G-s005

SC-013-D2SC00720G-s006

SC-013-D2SC00720G-s007
