# Supplementary material for: Heterotelechelic homopolymers mimicking high χ – ultralow N block copolymers with sub-2 nm domain size
Source: Chem Sci. 2022 Mar 14;13(14):4019–28. doi: 10.1039/d2sc00720g (PMC8985574; doi:10.1039/d2sc00720g)
Supplement: SC-013-D2SC00720G-s001 [file SC-013-D2SC00720G-s001.pdf]

Electronic Supplementary Material (ESI) for Chemical Science.
This journal is © The Royal Society of Chemistry 2022

Heterotelechelic homopolymers mimicking high χ – ultralow *N* block copolymers with sub-2 nm domain size

E. Hancox,[a] M. J. Derry,[b] M. J. Greenall,[c] S. Huband,[d] L. Al-Shok,[a] J. S. Town,[a] P. D. Topham*[b] and D. M. Haddleton*[a]

a. Department of Chemistry, University of Warwick, Coventry, CV4 7AL, UK. E-mail: d.m.haddleton@warwick.ac.uk

b. Aston Institute of Materials Research, Aston University, Birmingham, B4 7ET, UK. E-mail: p.d.topham@aston.ac.uk

c. School of Mathematics and Physics, University of Lincoln, Brayford Pool, Lincoln, LN6 7TS, UK

d. Department of Physics, University of Warwick, Coventry, CV4 7AL, UK

Supplementary Information

Experimental Section

Figures S1 – S32

References

Experimental Section

Materials

All solvents were of reagent grade and used directly. *Tert*-butyl acrylate was purchased from Sigma Aldrich and passed through a column of basic alumina to remove inhibitor prior to use. Tris[2-(dimethylamino)ethyl]amine (Me_6Tren) was synthesized following literature procedure and stored in a refrigerator.[1] α-bromoisobutyryl bromide, α-ethyl bromoisobutyrate (EBiB) and deuterated solvents were purchased from Sigma Aldrich and used as received. All other chemicals and reagents were purchased from Fisher Scientific and used as received.

Synthesis of perfluorooctyl bromoisobutyrate initiator (PFOBiB, F_{13})

DCM (60 ml), triethylamine (6.46×10^{-2} mol, 6.54 g) and 1H,1H,2H,2H-perfluorooctanol (5.36×10^{-2} mol, 19.52 g) were added to a 250 ml 3-necked round bottom flask (RBF) equipped with a constant pressure drop funnel. The RBF was purged with nitrogen and cooled in an ice-water bath containing NaCl and acetone. DCM (10 ml) containing α-bromoisobutyryl bromide (8.06×10^{-2} mol, 18.53 g) was placed in the constant pressure drop funnel and added dropwise to the reaction flask. The reaction was kept for 2 hrs in the ice-water bath followed by 36 hrs at 25 °C. The resulting solution was extracted (x4) in saturated aqueous $NaHCO_3$ solution and 1M NaOH solution, drying over anhydrous magnesium sulfate and recovery by filtration. Rotary evaporation of the solution resulted in a yellow liquid, which was further purified by flash column chromatography using DCM as the mobile phase and basic alumina as the stationary phase, followed by solvent evaporation under reduced pressure. Other volatiles were removed from the resulting yellow liquid in a vacuum oven at 25 °C overnight. Yield 78.4%. ^1H NMR (500 MHz, [D_1]$CDCl_3$): δ=4.49 (t, $^3J_{H,H}$=6 Hz, 2H; CO_2CH_2), 2.53 (tt, $^3J_{H,H}$=6, 18 Hz, 2H; CF_2CH_2), 1.94 ppm (s, 6H; $C(CH_3)_2$); ^{13}C NMR (125.8 MHz, $CDCl_3$): δ=171.5 (s; CO), 117.6 (tt, $^1J_{C,F}$=256, $^2J_{C,F}$=32 Hz; CF_2CH_2), 117.4 (qt, $^1J_{C,F}$=288, $^2J_{C,F}$=33 Hz; CF_3), 113.7-105.7 (m; $(CF_2)_4$), 58.0 (t, $^3J_{C,F}$=4 Hz; CH_2O), 55.1 (s; CBr), 30.7 (s; $C(CH_3)_2$); 30.5 ppm (t, $^2J_{C,F}$=22 Hz; CF_2CH_2); ^{19}F NMR (376.5 MHz, $CDCl_3$): -81.2 (t, $^3J_{C,F}$=10 Hz, 3F; CF_3), -113.6--113.9 (m, 2F; CF_3CF_2), -121.9--122.3 (m, 2F; $CF_3CF_2CF_2$), -122.9--123.3 (M, 2F; $CF_3(CF_2)_2CF_2$), -123.7--123.9 (m, 2F; $CF_3(CF_2)_3CF_2$), -126.3--126.6 ppm (m, 2F; $CF_3(CF_2)_4CF_2$).

Synthesis of perfluorodecyl bromoisobutyrate initiator (PFDBiB, F_{17})

Synthesis followed the same procedure as for PFOBiB, F_{13}, using 1H,1H,2H,2H-perfluorodecan-1-ol as starting material. Yield = 75.7%.

Synthesis of perfluorododecyl bromoisobutyrate initiator (PFDDBiB, F_{21})

Synthesis followed the same procedure as for PFOBiB, F_{13}, using 1H,1H,2H,2H-perfluorododecan-1-ol as starting material but with $CHCl_3$ as a solvent in place of DCM. Yield = 50.5%.

General procedure for photoinduced polymerisation - example target F_{13}-P*t*BA_{25}

$CuBr_2$ (0.02 eq., 2.5 mg) was dissolved in IPA (2 ml) with the aid of sonication, followed by addition of Me_6Tren (0.12 eq., 17.6 µl) and *t*BA (25 eq., 2 ml). The mixture was deoxygenated with nitrogen for

10 min before adding PFOBiB initiator (1 eq., 159 µl) and further deoxygenation for 5 min. The reaction was then left under an ultraviolet lamp (4 x 9W bulbs) for 12 hours. The resulting polymer was dissolved in minimum acetone, precipitated into deionised 1:1 MeOH:H_2O and passed through a short column of neutral alumina to remove remaining catalyst residues. Volatiles were removed and the product dried in a vacuum oven at 25 °C overnight. ^1H NMR was carried out in either $CDCl_3$ (Figure S6-Figure S8)

^1H NMR (300 MHz, $[D_1]CDCl_3$): δ=4.40-4.30 (m 2H; CO_2CH_2), 2.39-1.51 (m, 2H; CF_2CH_2), 1.94 ppm (s, 6H; $C(CH_3)_2$), 2.39-1.51 (m; $(CH_2CH)_n$), 1.45 (s; $((CH_3)_3)_n$), 1.23 (s, 2H; $COCH_2CH_2$), 1.15 ppm (s, 6H; $(CH_3)_2$). 99% conversion obtained by ^1H NMR. $Đ_{GPC(THF)}$ = 1.13, $M_{n,GPC}$ = 4300 g mol^{-1}.

General procedure for deprotection of P*t*BA – example target F_{13}-PAA_{25}

100 mg of F_{13}-P*t*BA_{25} was dissolved in 1 ml DCM. 1 ml TFA was added slowly and stirred at ambient temperature overnight. TFA was removed using by rotary evaporation and residual TFA was removed with the addition of acetone to form an azeotrope (x3). This caused the polymer to simultaneously precipitate and swell with the acetone. Acetone was added to dissolve a yellow impurity and removed by decanting (x3), the product was then dried under vacuum at 25°C overnight. Methanol was found as a good solvent for both segments and ^1H NMR was carried out in either CD_3OD or d^6-DMSO (Figure S9-Figure S11). Integration of backbone peaks approximately agreed with the values obtained from the F_n-P*t*BA_m spectra.

^1H NMR (400 MHz, $[D_1]CDCl_3$): δ=4.52-4.47 (m, 1H; CHBr), 4.40-4.28 (m, 2H; CO_2CH_2), 2.75-1.41 (m; $((CH_3)_3)_n$), 1.32 (s, 2H; $COCH_2CH_2$), 1.20 (d, 6H; $(CH_3)_2$). 99% conversion obtained by ^1H NMR. $M_{n,MALDI}$ = 2097 g mol^{-1}.

EBiB-PAA_m

Synthesised following the general procedure for photoinduced polymerisation, substituting the fluorocarbon initiator for α-ethyl bromoisobutyrate (EBiB). Deprotection follows the general procedure for deprotection of P*t*BA.

Film preparation/Annealing

3 ml of a saturated solution of F_n-PAA_m in methanol was placed in a small PTFE boat. This boat and a separate vial of methanol were placed under a beaker and left to evaporate for 48 hours. A thick polymer film remained in the PTFE boat. These films were then either thermally annealed at a static temperature of 120 °C for 24 hours (static temperature SAXS) and cooled slowly to room temperature or heated to 150 °C gradually while performing SAXS measurements (time-resolved during thermal annealing measurements). See "*Time-resolved during thermal annealing measurements*" in Instrumental Section for more details.

Instrumental Section

^{1}**H NMR.** NMR spectra were recorded on Bruker HD-300 MHz, HD-400 MHz spectrometers and a Bruker AV III-500 MHz HD spectrometer equipped with a cryoprobe. Monomer conversion was calculated by comparison of vinyl protons (6.4-5.6 ppm) with $(CH_3)_3$ protons (1.45 ppm) of P*t*BA.

Size Exclusion Chromatography

THF - Standard. Agilent Infinity II MDS instrument equipped with differential refractive index (DRI). The system was equipped with 2 x PLgel Mixed C columns (300 x 7.5 mm) and a PLgel 5 µm guard column. The eluent used was THF with 2 % TEA (triethylamine) and 0.01 % BHT (butylated hydroxytoluene) additives. Samples were run at 1 ml/min at 30°C. Narrow molecular weight poly(methyl methacrylate) (11 narrow standards between 2210000-1010 Da) and polystyrene standards (12 narrow standards between 364000-160 Da (Agilent EasiVials) were used for calibration. Analyte samples were filtered through a GVHP membrane with 0.22 µm pore size before injection. Respectively, experimental molar mass (M_n) and dispersity ($Đ$) values of synthesised polymers were determined by conventional calibration using Agilent GPC/SEC software.

MALDI-ToF-MS

Samples (of lower molecular weight) for MALDI ToF analysis were dissolved at 10 mg/ml in THF with 1 mg/ml of sodium iodide. 10 µl of this sample mixture was then mixed with 10 µl matrix solution of 40 mg/ml trans-2-[3-(4-tert-butylphenyl)-2-methyl-2-propenylidene]malononitrile (DCTB) in THF and 1 mg/ml of sodium iodide. Samples (of higher molecular weight) for MALDI ToF analysis were dissolved at 10 mg/ml in H_2O with 1 mg/ml of sodium iodide. 10 µl of this sample mixture was then mixed with 10 µl matrix solution of 40 mg/ml 2,5-Dihydroxybenzoic acid (DHB) in MeOH and 1 mg/ml of sodium iodide. 0.5 µl of the resulting solution was then spotted on a 384 ground steel multi-target plate, using a dried droplet methodology. The dried crystal structure was then analysed using a Bruker Autoflex, equipped with a 337 nm N_2 laser, operating in reflectron positive mode with an ion source voltage of 19 kV. Data analysis was then carried out on Bruker fleXanalysis and mMass.

End group analysis is discussed in more detail in our previous work.[2] Distributions include Br termination, H termination, H termination with a sodium acrylate unit, a cyclised end group and a cyclised end group with a sodium acrylate unit (Figure S17). The presence of a sodium acrylate unit arises from substitution of a proton during the MALDI preparation with a sodium ion and is not representative of the bulk sample.

X-Ray Scattering

Single data acquisition measurements. X-ray scattering measurements were made using a Xenocs Xeuss 2.0 equipped with a micro-focus Cu Kα source collimated with Scatterless slits. For static measurements, samples were mounted between two sticky Kapton windows. Small-angle X-ray scattering (SAXS) was measured using a Pilatus 300k detector with a pixel size of 0.172 mm x 0.172 mm. The detector was translated vertically and the images combined to form a virtual detector to obtain data over a wider q range. The magnitude of the scattering vector (q) is given by $q = 4\pi\sin\theta/\lambda$, where 2θ is the angle between the incident and scattered X-rays and λ is the wavelength of the incident X-rays. The distance between the detector and the sample was calibrated using silver behenate ($AgC_{22}H_{43}O_2$), giving a value of 0.339(5) m, corresponding to a q range of 0.035 Å^{-1} to

1.66 Å^{-1}. Radial integration as a function of *q* was performed on the 2D scattering profiles and the resulting data corrected for the absorption and background from the sample holder.

Time-resolved SAXS during thermal annealing measurements. These experiments were carried out in a similar way to the single data acquisition measurements, with a Linkam HFSX 350 being used to control the temperature of samples mounted between Kapton sheets. Samples were heated and cooled at a rate of 0.5 °C/min while making 1 min data collections. After every 5 measurements the transmitted intensity was measured for making corrections.

Liquid initiator measurements. These were carried out in a similar way to the single data acquisition measurements but the samples were mounted in 1 mm diameter borosilicate glass capillaries instead. The Kapton peak is present due to the Kapton windows used in the main SAXS chamber. These windows are necessary to allow air into the chamber for liquid samples.

Thermal Analysis

Differential Scanning Calorimetry (DSC). Data for F_{13}-PAA_m polymers were obtained using a Mettler-Toledo DSC1 with autosampler under nitrogen, samples were heated and cooled between 25-180 ^oC at 10 ^oC per minute. Heating cycles were repeated 3 times. Data for F_{17}-PAA_m and F_{21}-PAA_m polymers were obtained using PEDSC6000 with intracooler, samples were heated and cooled between 0-200 ^oC at 10 ^oC per minute. Heating cycles were repeated 3 times.

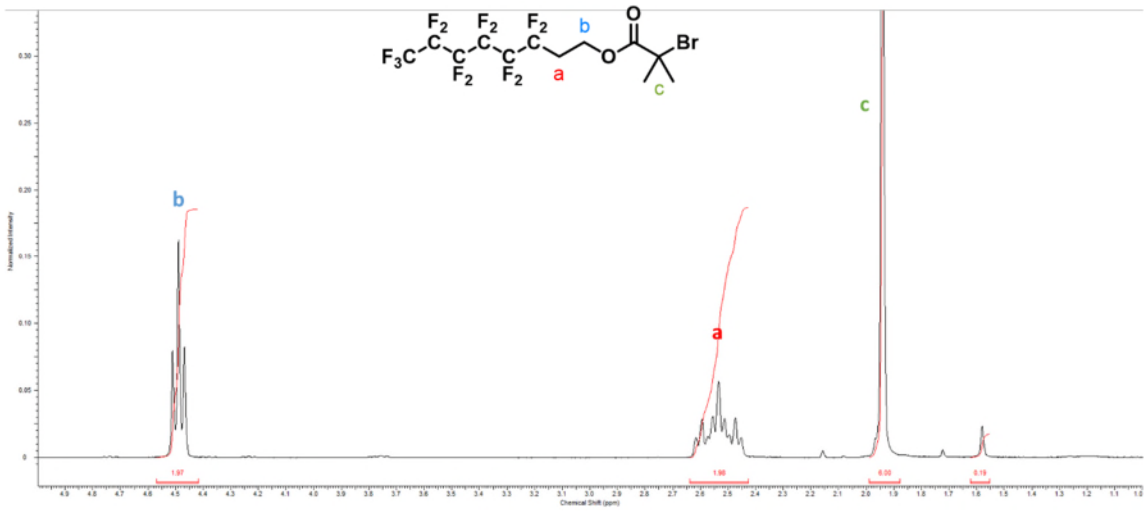

Figure S1. ^{1}H NMR spectrum (500 MHz, $CDCl_3$) of F_{13} initiator in $CDCl_3$ with assignment.

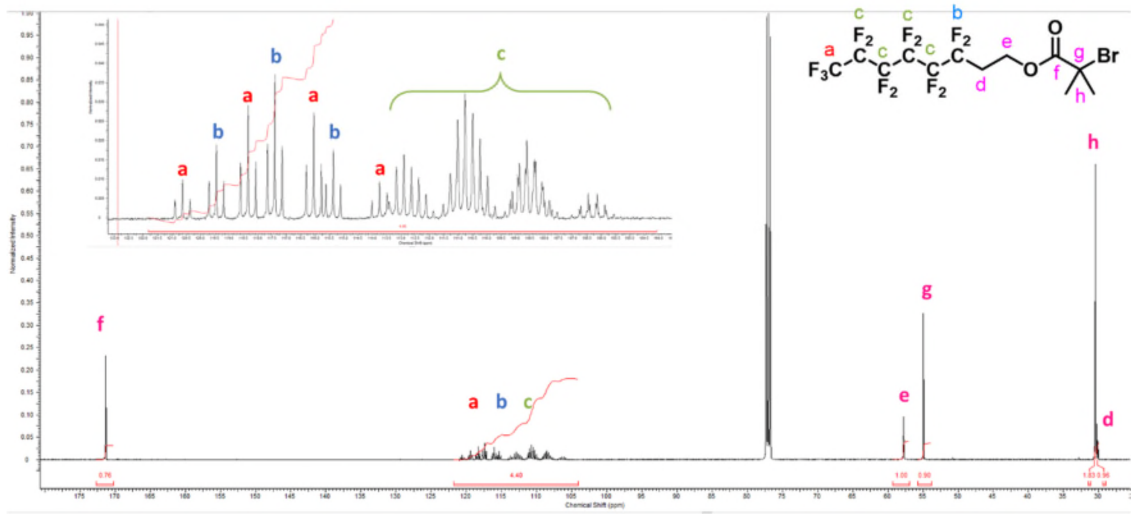

Figure S2. ^{13}C NMR spectrum (500 MHz, $CDCl_3$) of F_{13} initiator in $CDCl_3$. Insert shows expansion of region 122 – 104 ppm.

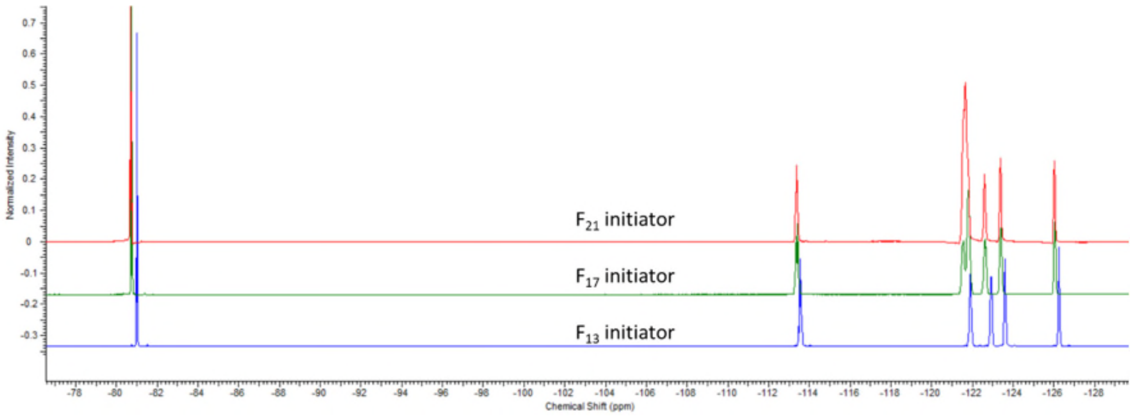

Figure S3. ^{19}F NMR (coupled) spectrum (400 MHz, $CDCl_3$) of F_{13}, F_{17} & F_{21} initiators in $CDCl_3$.

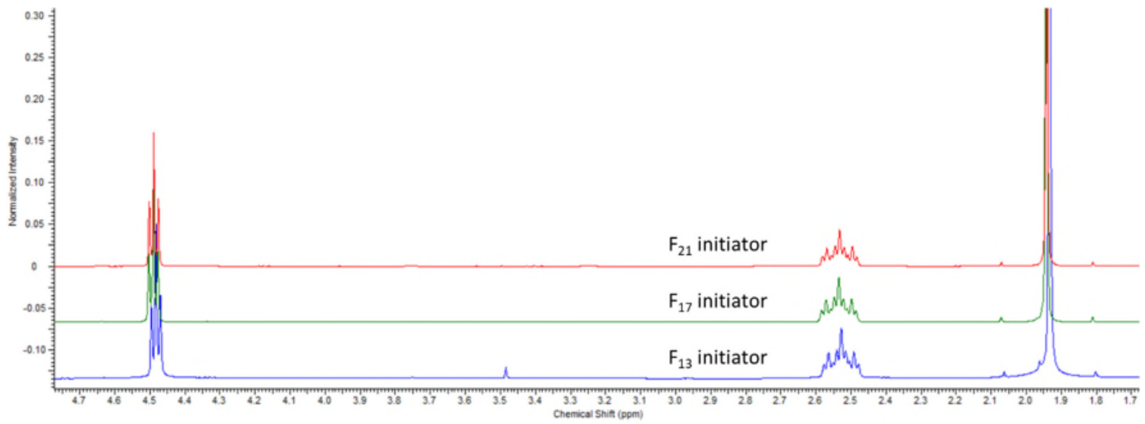

Figure S4. ^{1}H NMR spectrum (500 MHz, $CDCl_3$) of F_{13}, F_{17} & F_{21} initiators in $CDCl_3$.

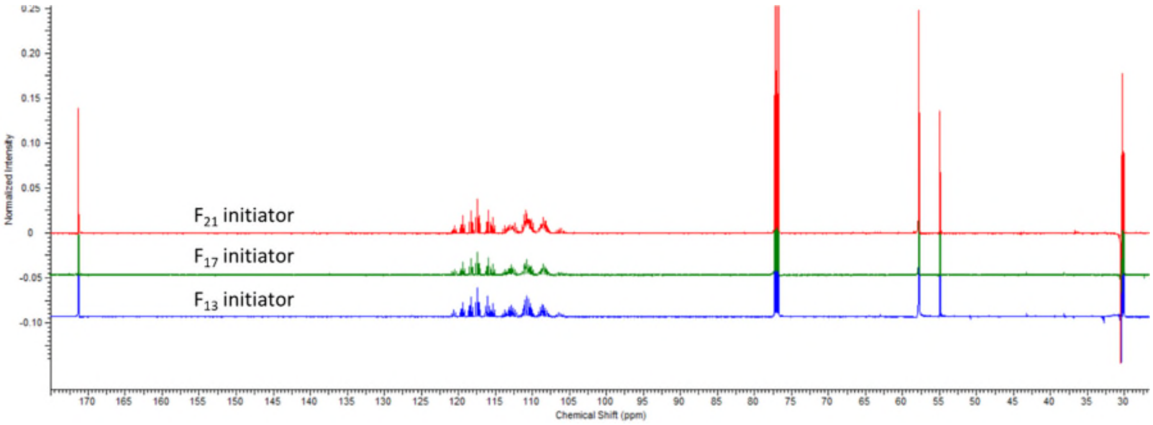

Figure S5. ^{13}C (ATP) NMR spectrum (500 MHz, $CDCl_3$) of F_{13}, F_{17} & F_{21} initiators in $CDCl_3$.

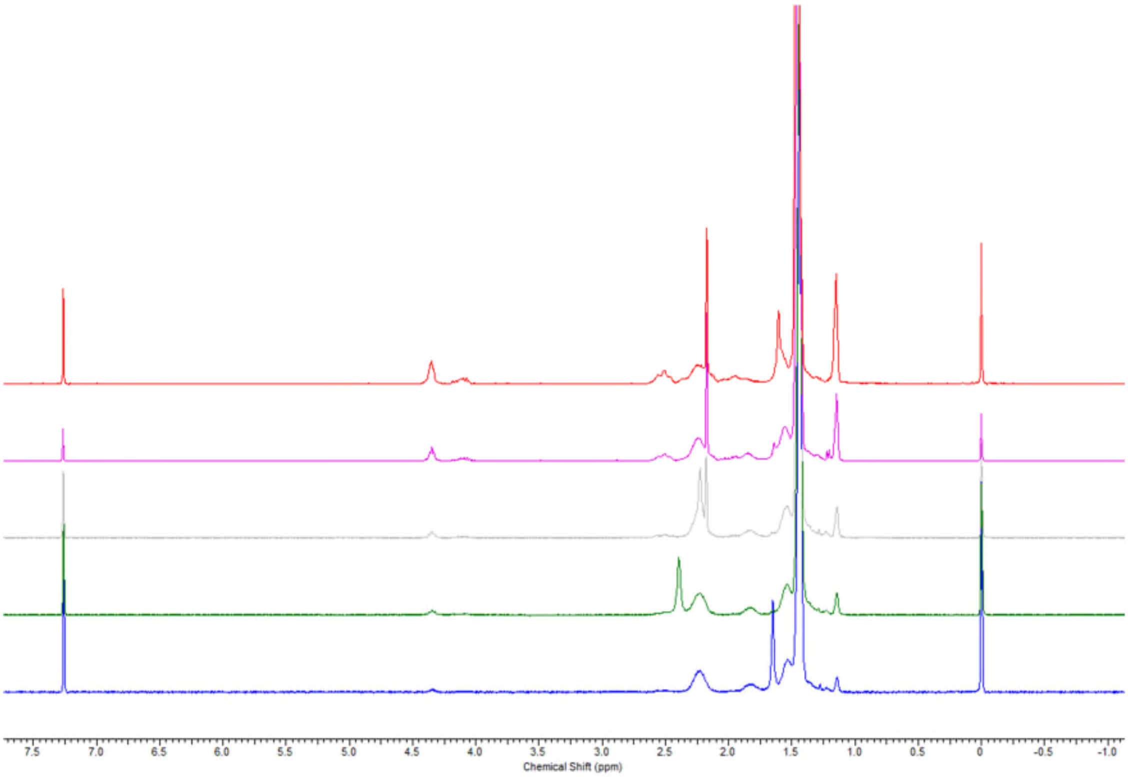

Figure S6. ^{1}H NMR spectrum (400 MHz, $CDCl_3$) of F_{13}-P*t*BA_m in $CDCl_3$. m = 6, 12, 17, 22, 27 from top to bottom.

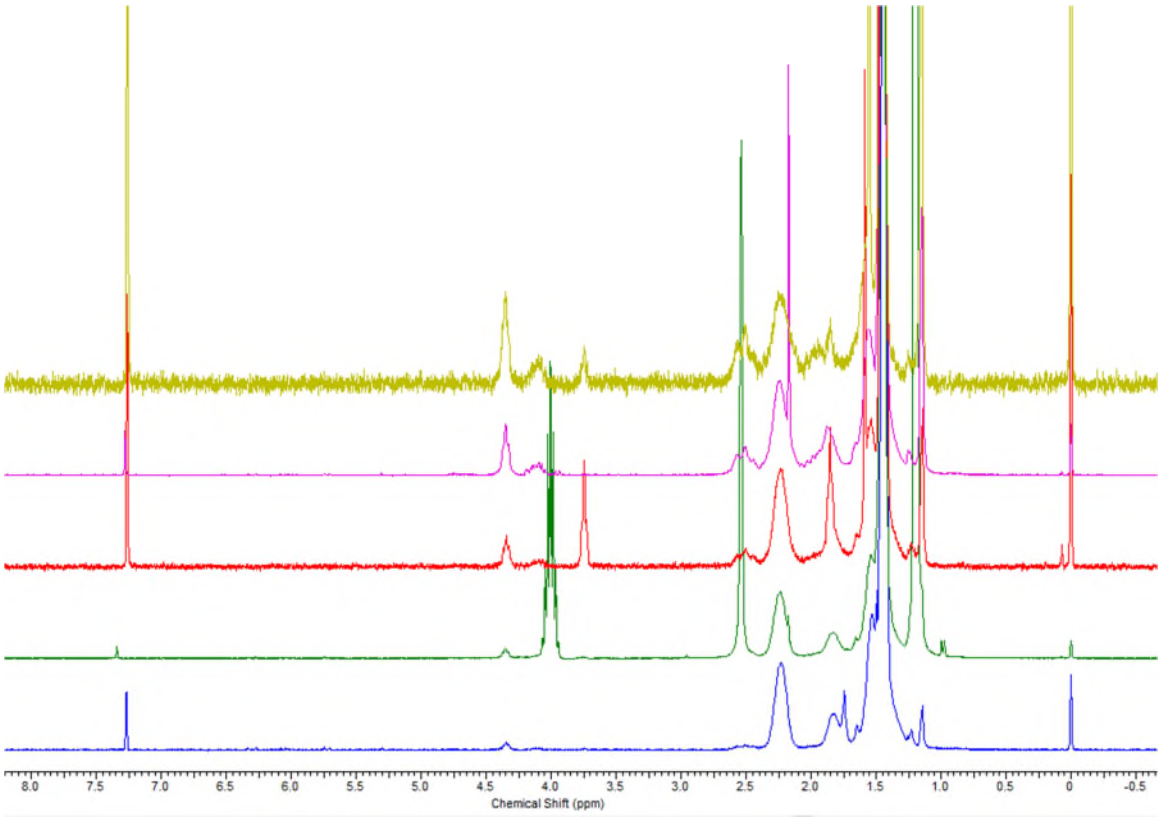

Figure S7. ^{1}H NMR spectrum (400 MHz, $CDCl_3$) of F_{17}-P*t*BA_m in $CDCl_3$. m = 6, 11, 17, 23, 30 from top to bottom.

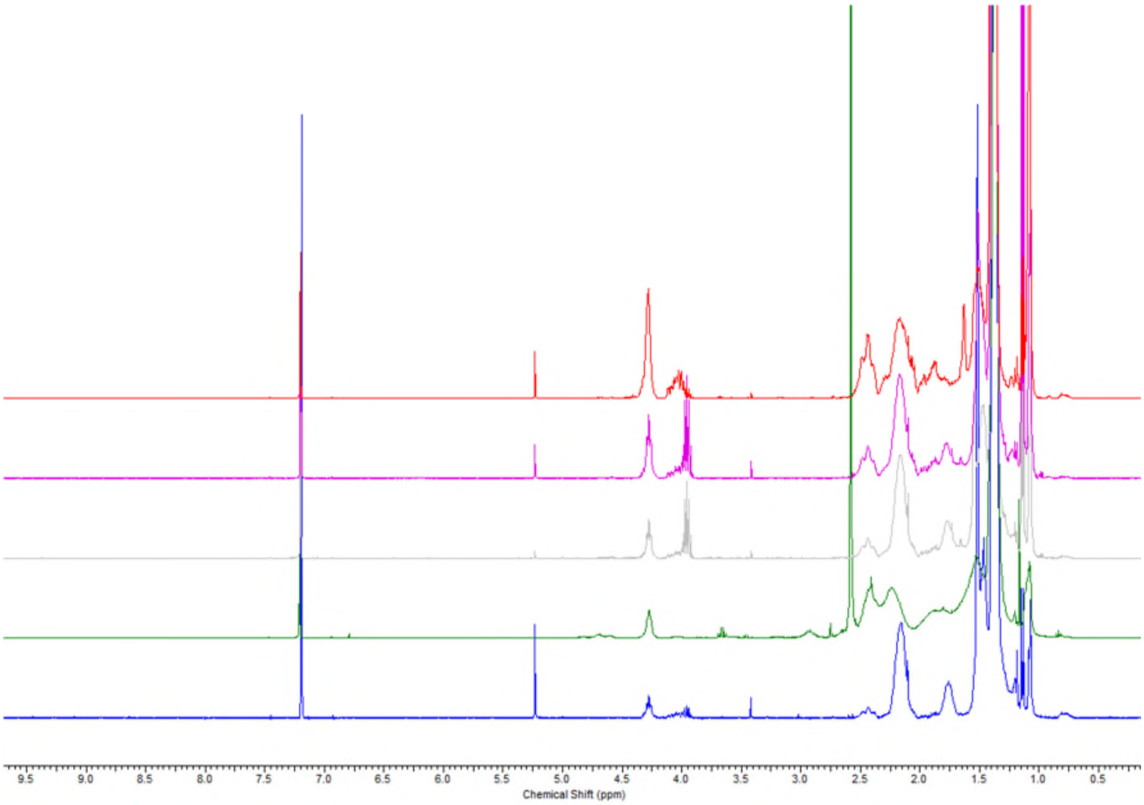

Figure S8. ^{1}H NMR spectrum (400 MHz, $CDCl_3$) of F_{21}-P*t*BA$_m$ in $CDCl_3$. m = 5, 10, 16, 20, 24 from top to bottom.

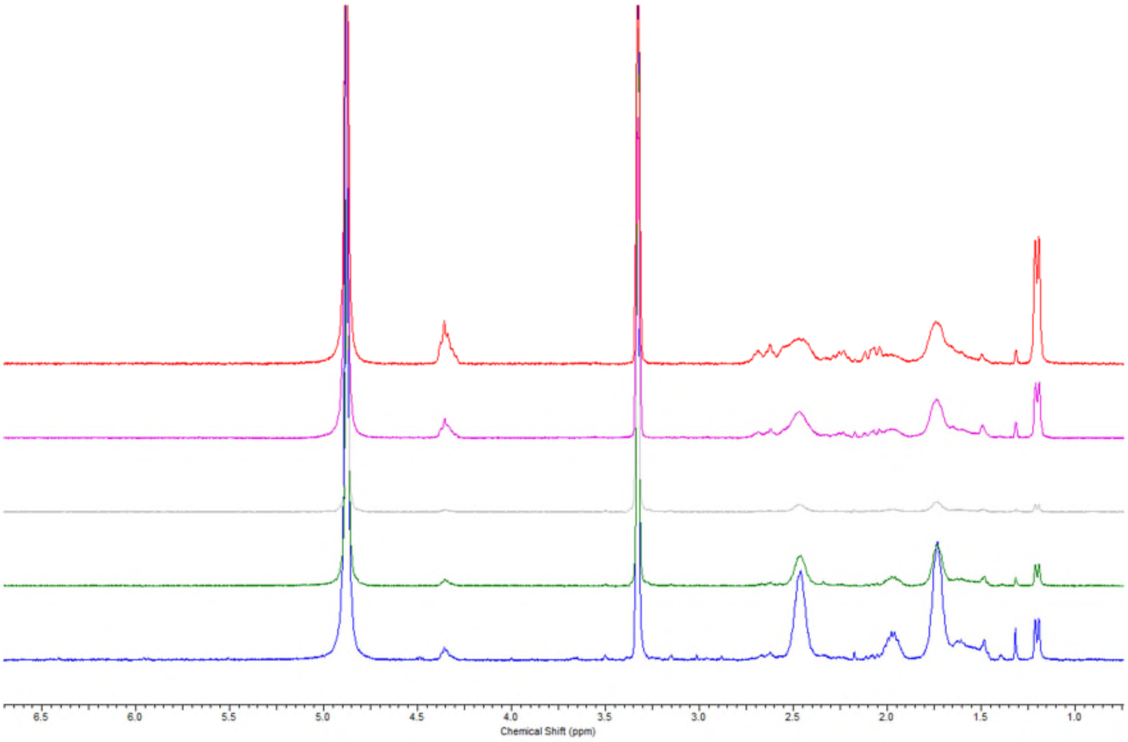

Figure S9. ^{1}H NMR spectrum (400 MHz, $CDCl_3$) of F_{13}-PAA$_m$ in MeOD. m = 6, 12, 17, 22, 27 from top to bottom.

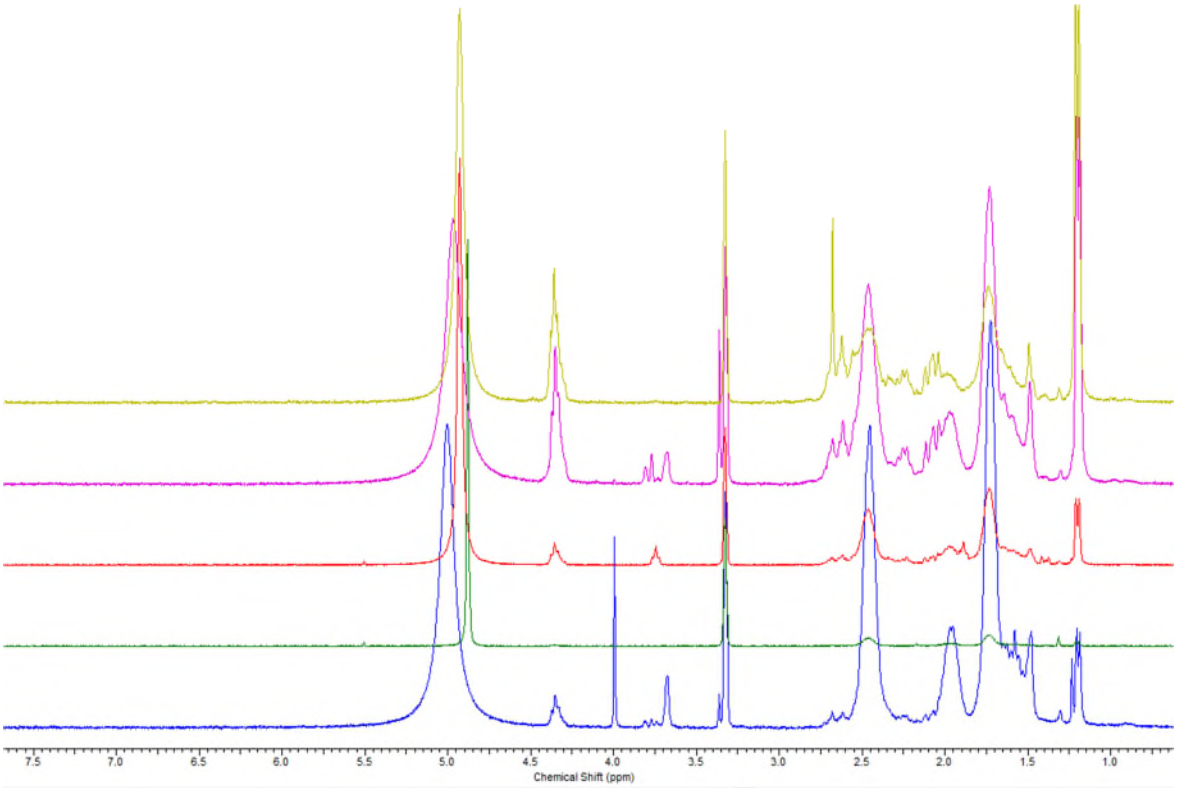

Figure S10. ^{1}H NMR spectrum (400 MHz, $CDCl_3$) of F_{17}-PAA_m in MeOD. m = 6, 11, 17, 23, 30 from top to bottom.

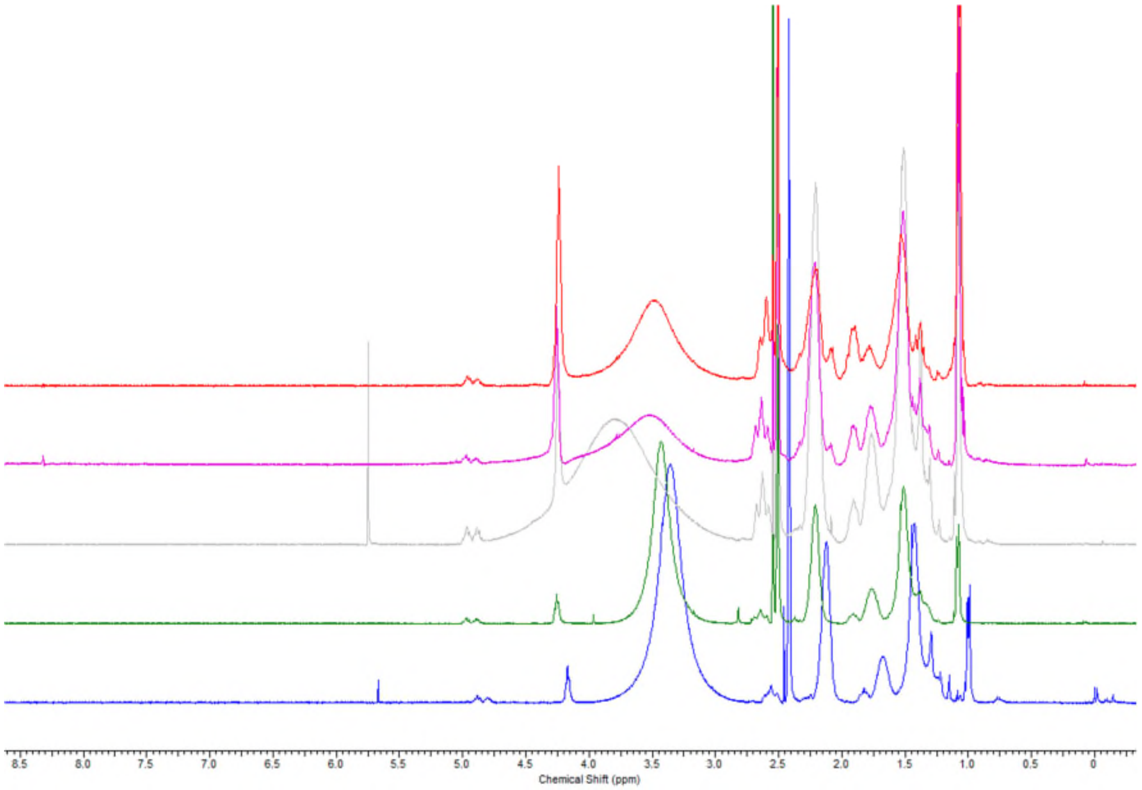

Figure S11. ^{1}H NMR spectrum (400 MHz, $CDCl_3$) of F_{21}-PAA_m in d^6-DMSO. m = 5, 10, 16, 20, 24 from top to bottom.

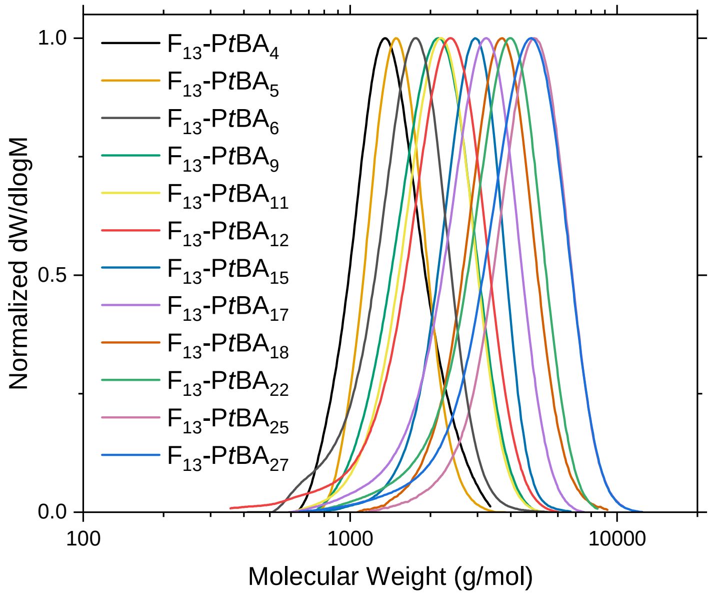

Figure S12. GPC traces of F_{13}-P*t*BA_m recorded in THF eluent.

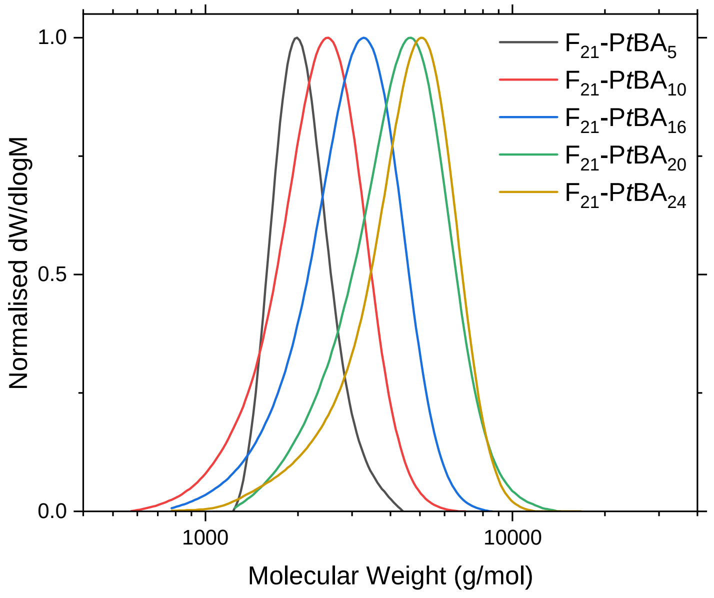

Figure S13. GPC traces of F_{21}-P*t*BA$_m$ recorded in THF eluent.

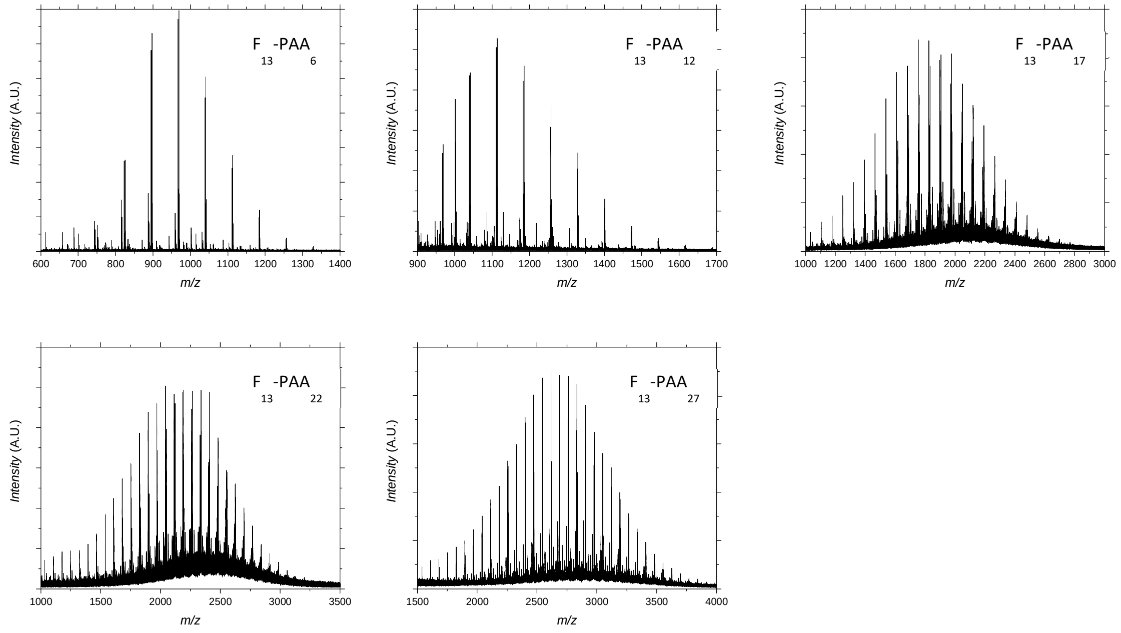

Figure S14. MALDI-ToF-MS spectra for F_{13}-PAA$_m$ polymers.

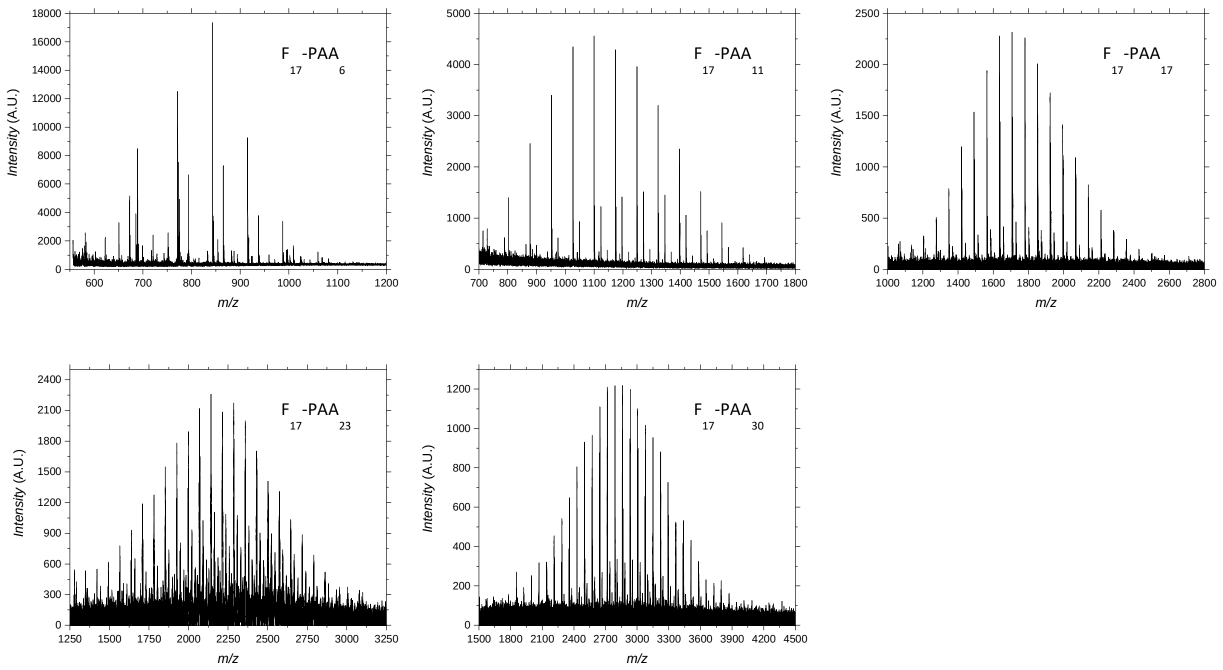

Figure S15. MALDI-ToF-MS spectra for F_{17}-PAA_m polymers.

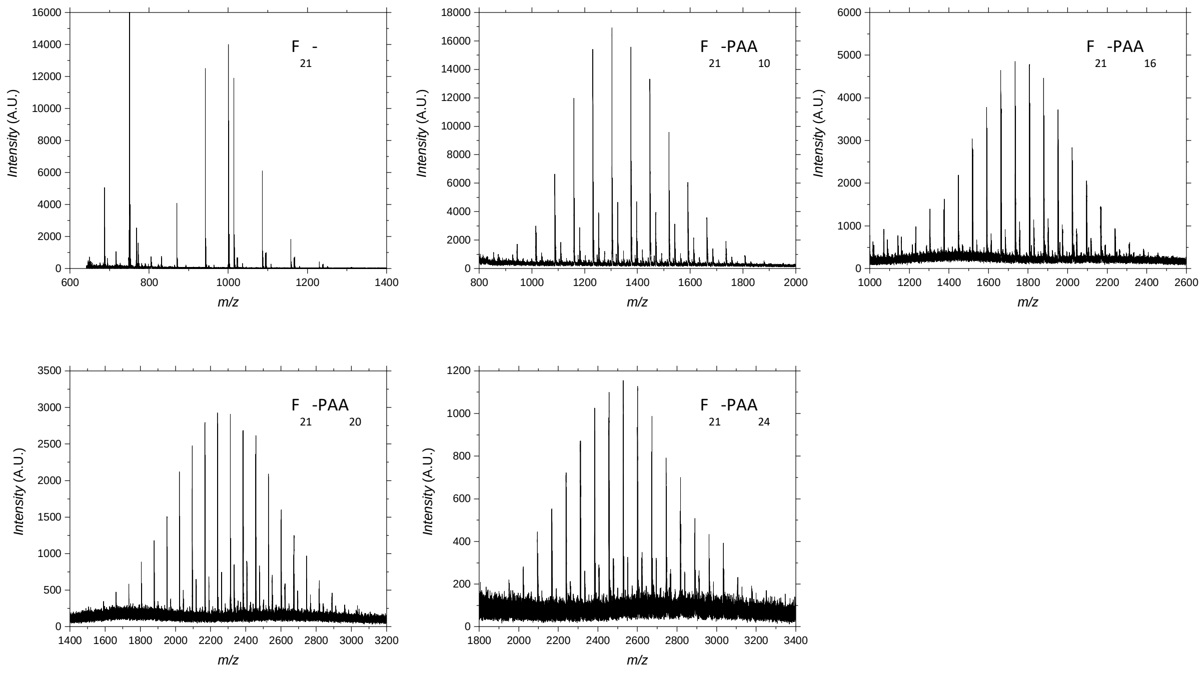

Figure S16. MALDI-ToF-MS spectra for F_{21}-PAA_m polymers.

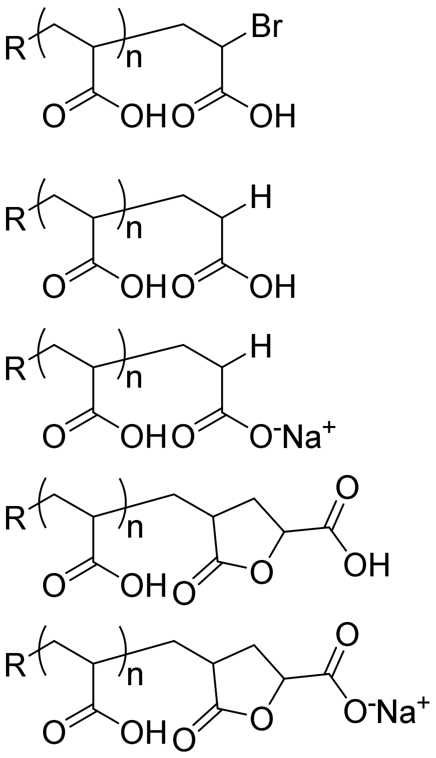

Figure S17. End group structures determined from MALDI spectra for all F_n-PAA_m polymers.

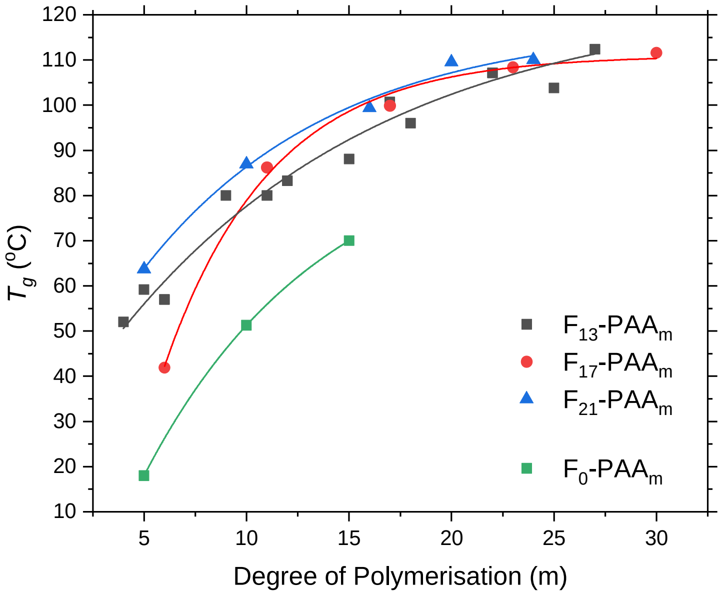

Figure S18. T_g determined from DSC traces vs DP for F_n-PAA_m polymers. F_0 is given for EBiB initiated polymers.

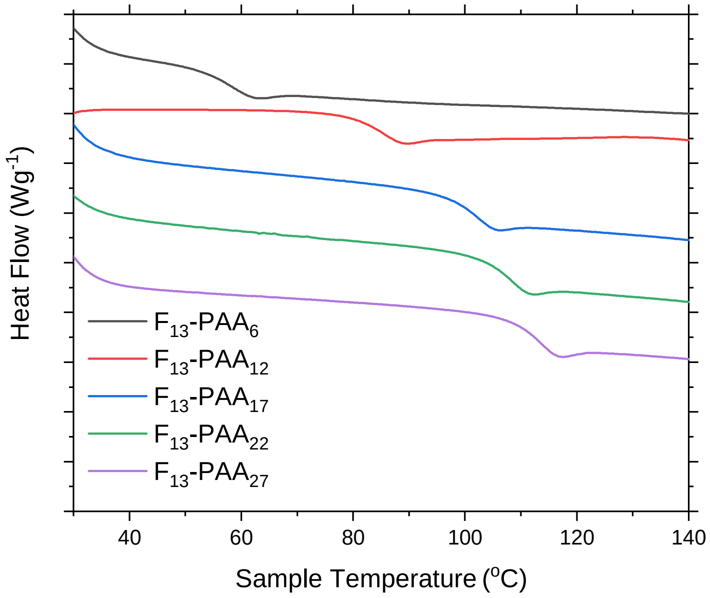

Figure S19. Differential Scanning Calorimetry (DSC) traces for F_{13}-PAA_m polymers. Samples were heated from 25 to 180 °C at 10 °C / minute. Traces shown are from the third heating cycle.

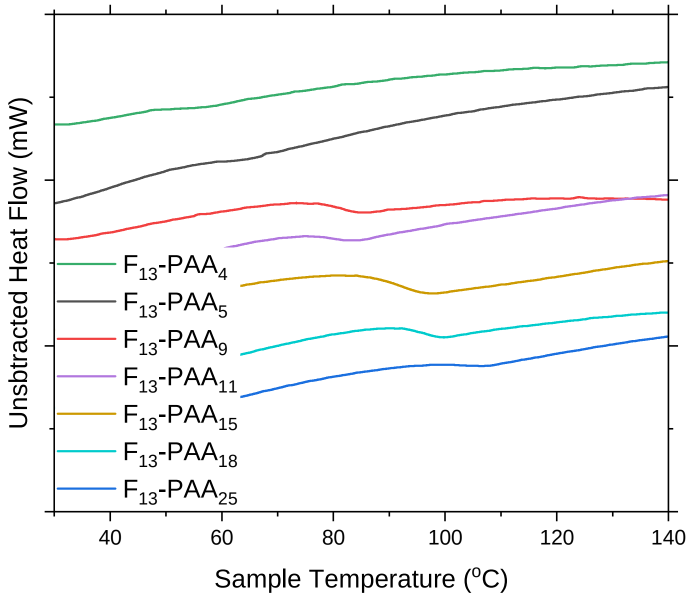

Figure S20. Differential Scanning Calorimetry (DSC) traces for F_{13}-PAA_m polymers. Samples were heated from 25 to 180 °C at 10 °C / minute. Traces shown are from the third heating cycle.

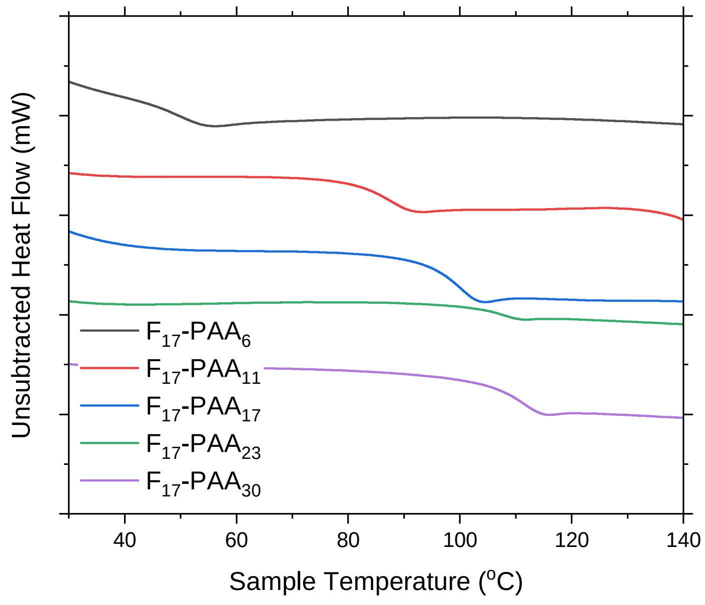

Figure S21. Differential Scanning Calorimetry (DSC) traces for F_{17}-PAA_m polymers. Samples were heated from 0 to 200 °C at 10 °C / minute. Traces shown are from the third heating cycle.

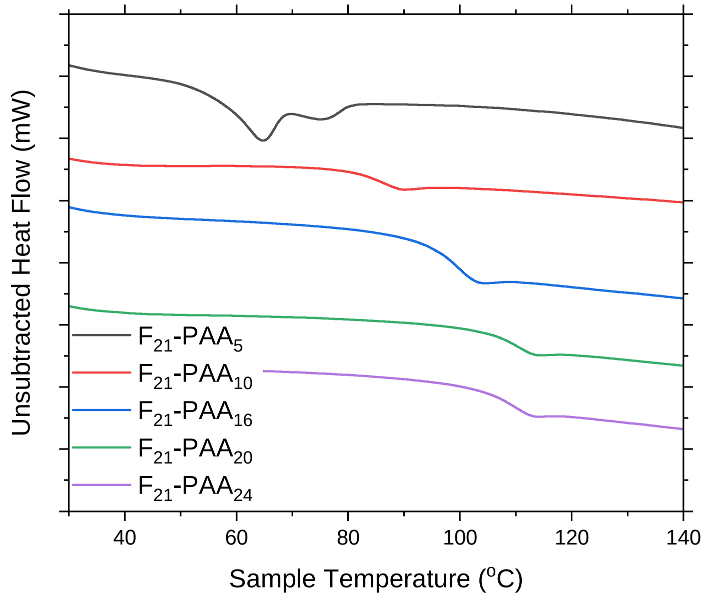

Figure S22. Differential Scanning Calorimetry (DSC) traces for F_{21}-PAA_m polymers. Samples were heated from 0 to 200 °C at 10 °C / minute. Traces shown are from the third heating cycle.

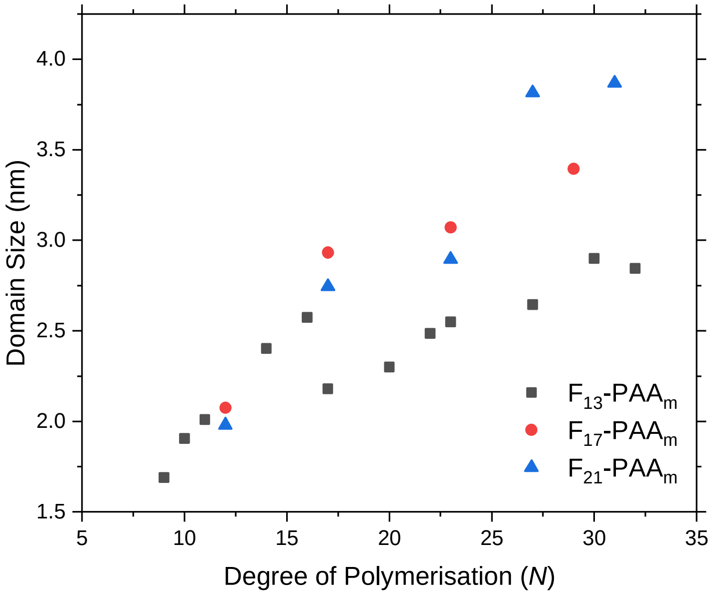

Figure S23. Domain Size (*d**) vs Total Degree of Polymerisation (*N*) single data acquisition measurements of all F_n-PAA_m sets.

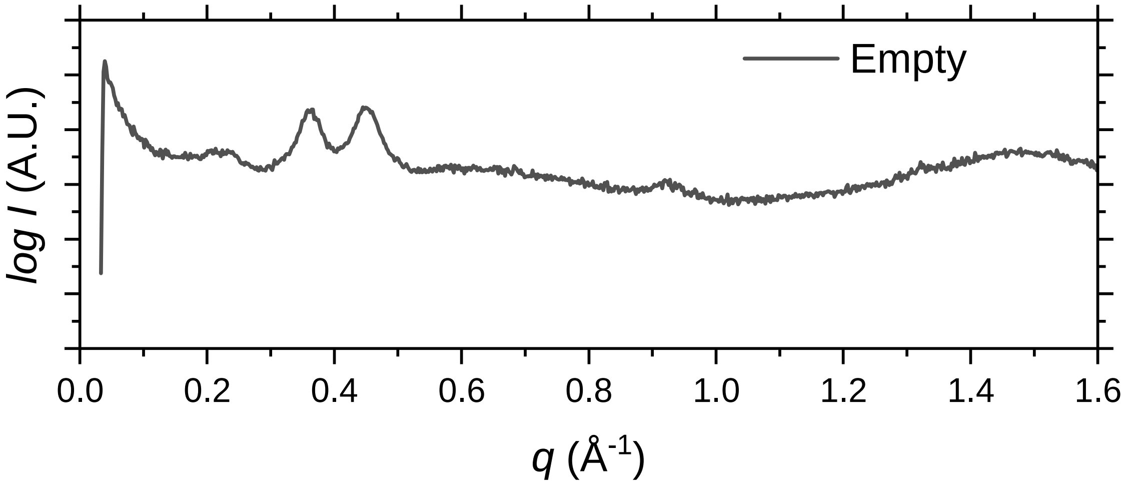

Figure S24. SAXS profile for a blank in liquid capillary measurements. N.B. The Kapton peaks are present due to the Kapton windows used in the main SAXS chamber. These windows are necessary to allow air into the chamber for liquid samples.

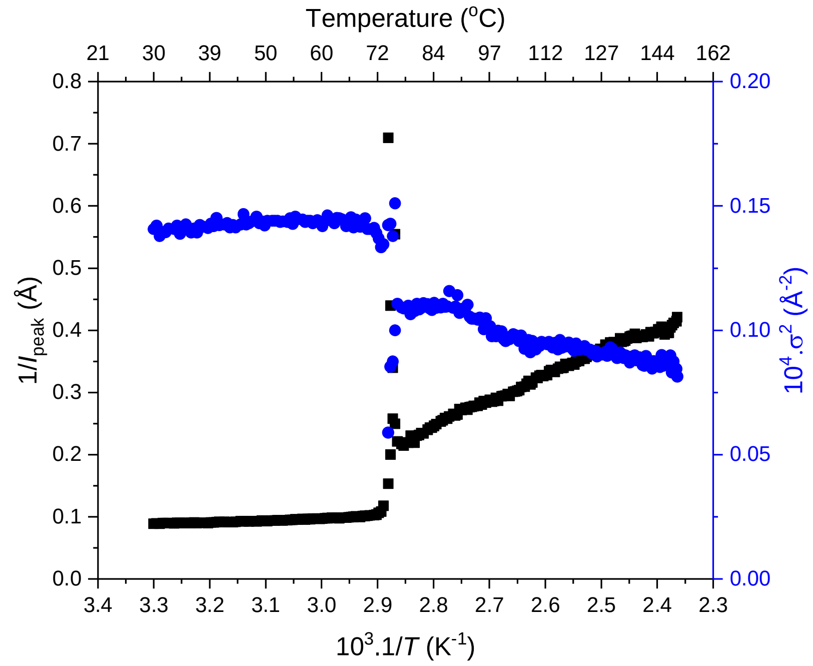

Figure S25. 1/peak intensity and FWHM^2 (σ^2) from time-resolved SAXS heating cycle vs 1/temperature for F_{21}-PAA_5. Transitions at 73.6 °C and 95.7 °C (T_{ODT}).

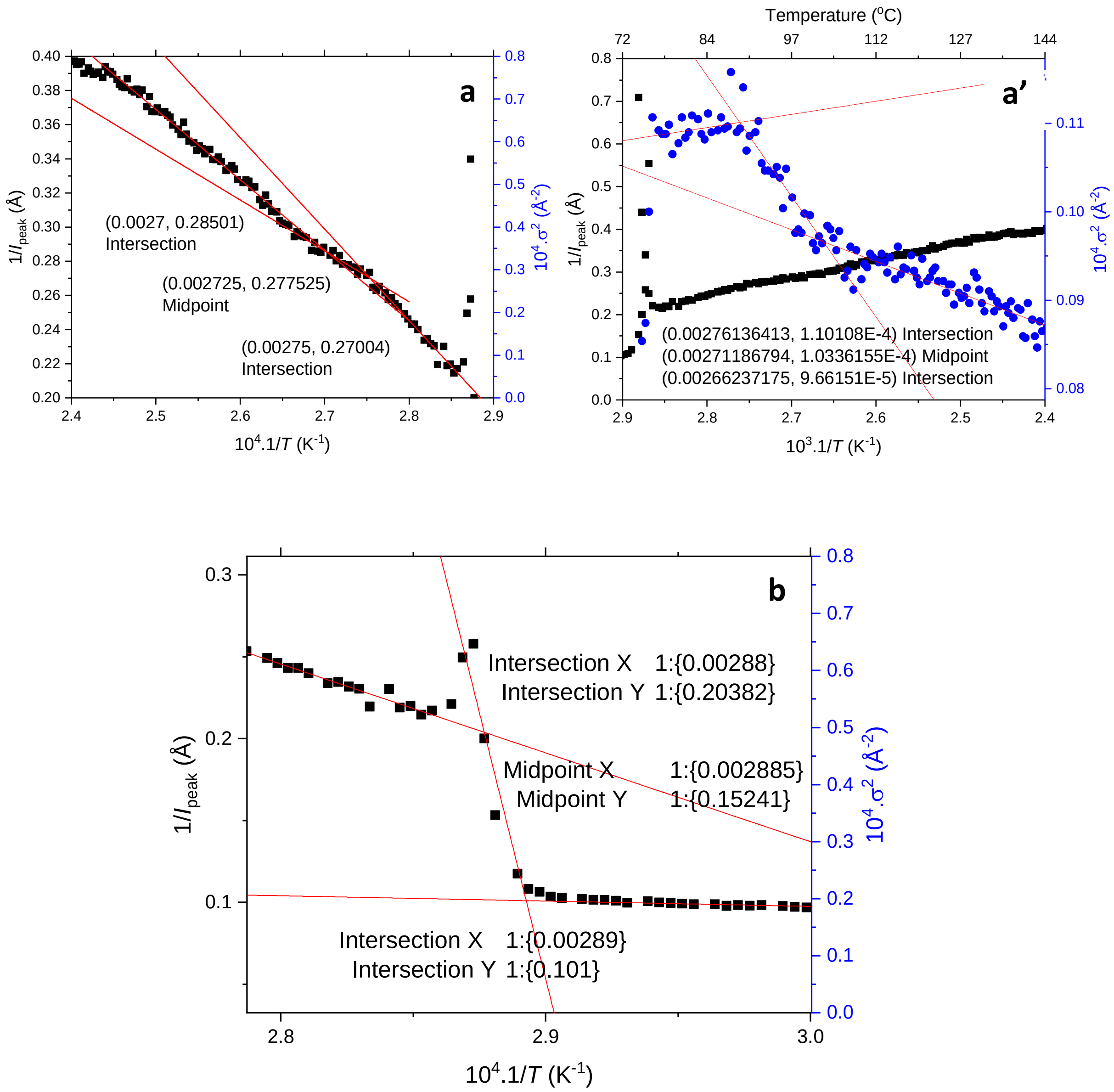

Figure S26. Demonstration of calculation of phase transition temperatures (from Figure S25). The midpoint of two intersections from linear fits before, during and after the transition gives the transition temperature. **a** = transition at 94.0 °C, **a'** = transition at 94.0 °C calculated from FWHM^2 (σ^2) (95.7 °C), **b** = transition at 73.6 °C.

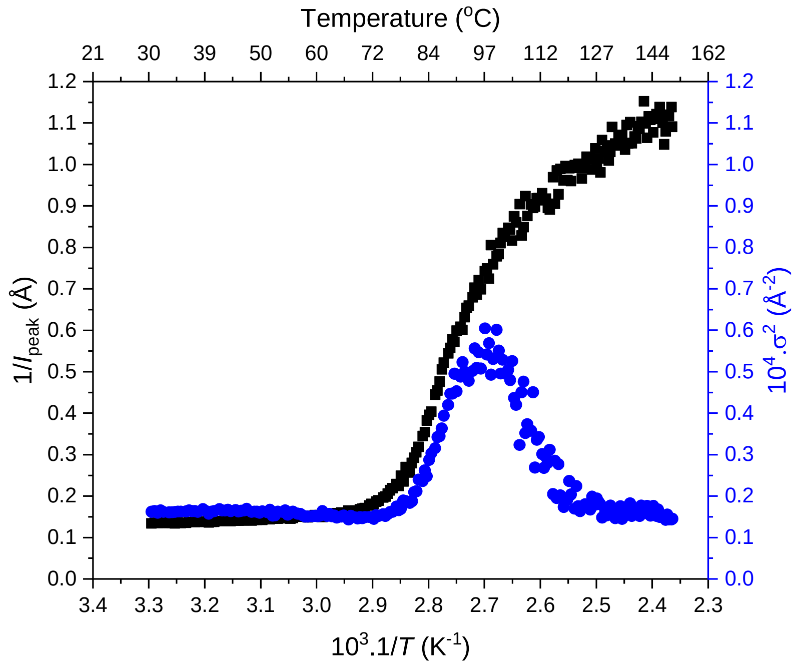

Figure S27. 1/peak intensity and FWHM[2] (σ^2) from time-resolved SAXS heating cycle vs 1/temperature for F_{21}-PAA_{10}. T_{OOT} = 72-80 °C. T_{ODT} = 87.4 °C.

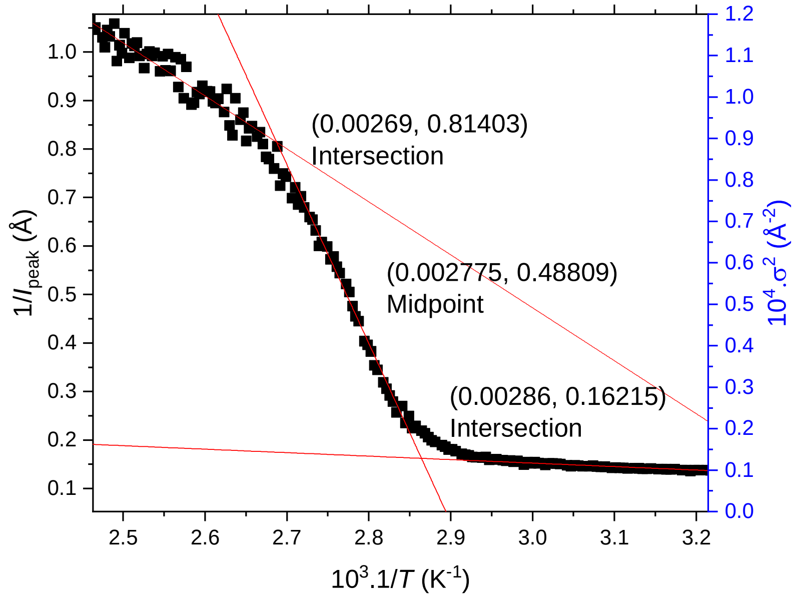

Figure S28. Demonstration of calculation of phase transition temperatures (from Figure S27). The midpoint of two intersections from linear fits before, during and after the transition gives the transition temperature.

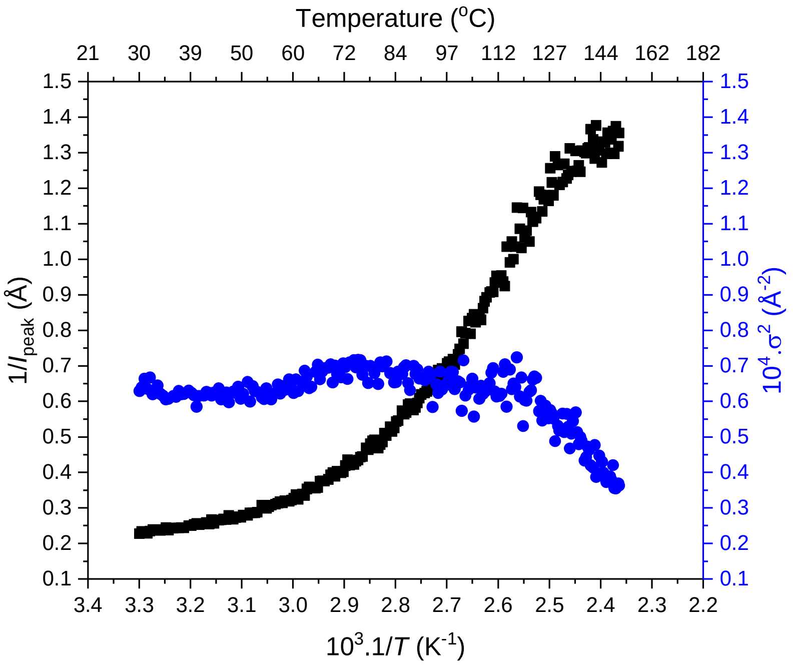

Figure S29. 1/peak intensity and FWHM^2 (σ^2) from time-resolved SAXS heating cycle vs 1/temperature for F_{21}-PAA_{16}. T_{ODT} = 102.9 °C.

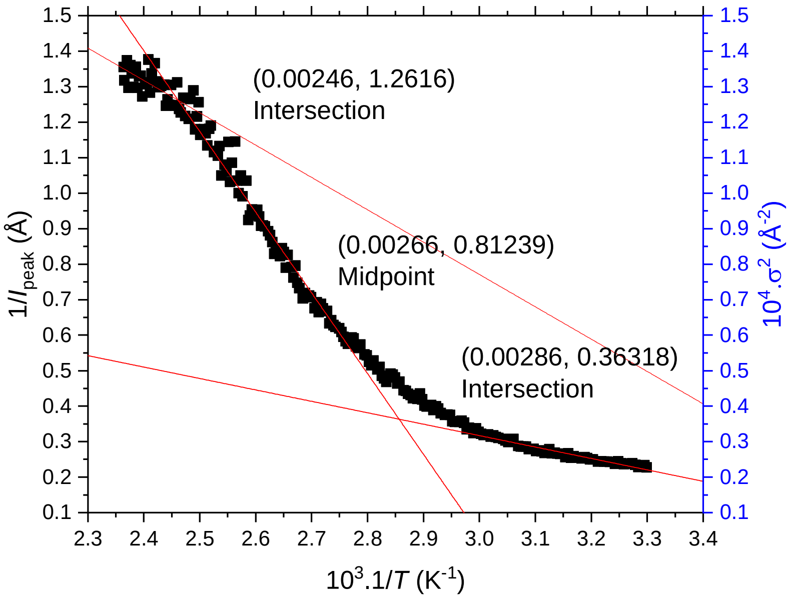

Figure S30. Demonstration of calculation of phase transition temperatures (from Figure S29). The midpoint of two intersections from linear fits before, during and after the transition gives the transition temperature.

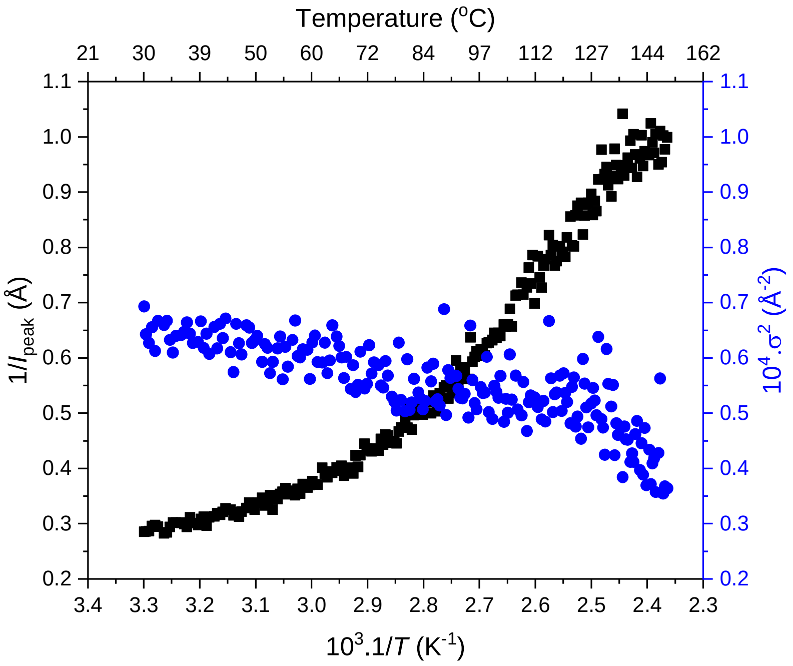

Figure S31. 1/peak intensity and FWHM^2 (σ^2) from time-resolved SAXS heating cycle vs 1/temperature for F_{21}-PAA_{20}. T_{ODT} = 105.8 °C.

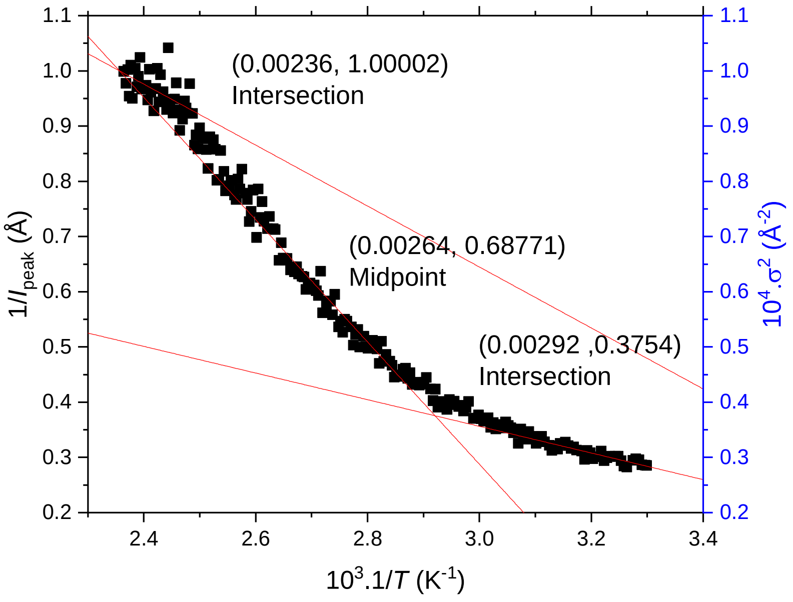

Figure S32. Demonstration of calculation of phase transition temperatures (from Figure S31). The midpoint of two intersections from linear fits before, during and after the transition gives the transition temperature.

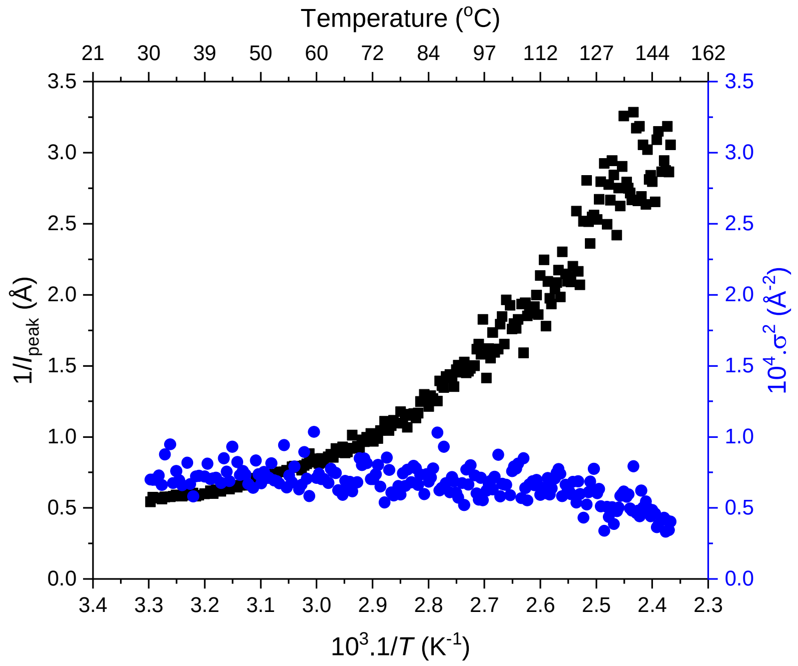

Figure S33. 1/peak intensity and FWHM^2 (σ^2) from time-resolved SAXS heating cycle vs 1/temperature for F_{21}-PAA_{24}. No T_{ODT}.

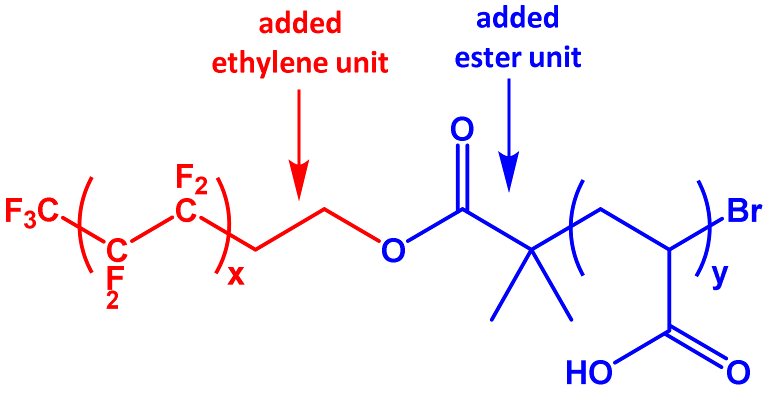

Figure S34. Chemical structure of the polymers in this work, where the colours indicate the chemical groups attributed to the separate segments (fluorinated segment in red and PAA segment in blue).

The volume fractions of each segment were calculated to account for the bridging unit between the 'polytetrafluoroethene' (PTFE) and PAA segments. The degree of polymerisation (DP) of the fluorinated segment was counted as number of tetrafluoroethylene (TFE) repeat units in the fluorinated initiator used *i.e.* 3, 4 or 5 for F_{13}, F_{17} or F_{21}, respectively. An additional unit was added to the DP of the PTFE segment in the volume fraction calculations to account for the adjacent ethylene unit which is approximated to have the same volume as a TFE repeat unit, taking the DP of the PTFE unit to 4, 5 or 6. The DP of the PAA differed in each polymer. When calculating the volume fractions of this segment, an additional repeat unit was added to account for the adjacent ester unit and approximated to have the same volume as the AA units. Figure S29 shows how the polymer was compartmentalised for the volume fraction calculations. The PTFE block with its adjacent ethylene unit is shown in red and the PAA segment with the neighbouring ester unit in blue.

The volume fractions were calculated using the following equations:

$$f_{PAA} = \frac{{(N_{PAA} \times Mr_{AA})}/{\rho_{PAA}}}{\left({(N_{PAA} \times Mr_{AA})}/{\rho_{PAA}}\right)+\left({(N_{PTFE} \times Mr_{TFE})}/{\rho_{PTFE}}\right)} \qquad f_{PTFE} = 1 - f_{PAA}$$

where N_{PAA} and N_{PTFE} are the degrees of polymerisation of the PAA and PTFE segments, respectively, each with an additional unit added to account for the bridging unit. $M_{r,AA}$ is the molar mass of acrylic acid (72.06 g mol^{-1}) and $M_{r,TFE}$ is the molar mass of tetrafluoroethene (100.02 g/mol). ρ_{PAA} and ρ_{PTFE} are the densities of PAA and PTFE which are taken as 1.41 and 2.2 g/mL, respectively.

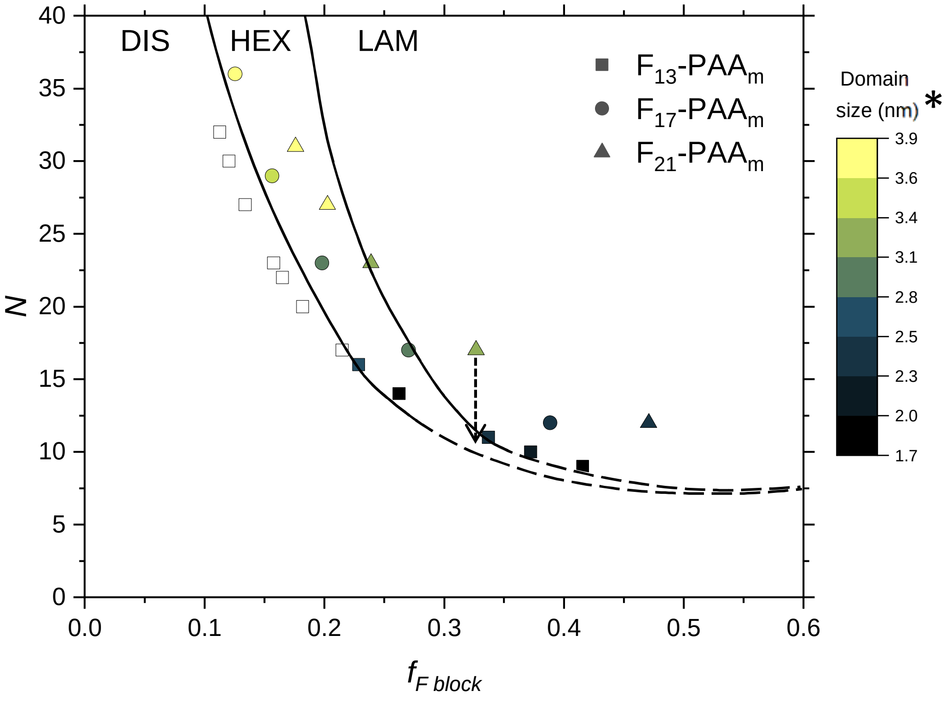

Figure S35. Phase diagram for F_n-PAA_m polymers, including data obtained in previous research.[2] Data point at N = 17 (F_{21}-PAA_{10}) showed an order-order transition from LAM (lamellae) to HEX (hexagonally packed cylinders) during time-resolved measurements. Dotted arrow indicates the data point shift across the phase boundary.

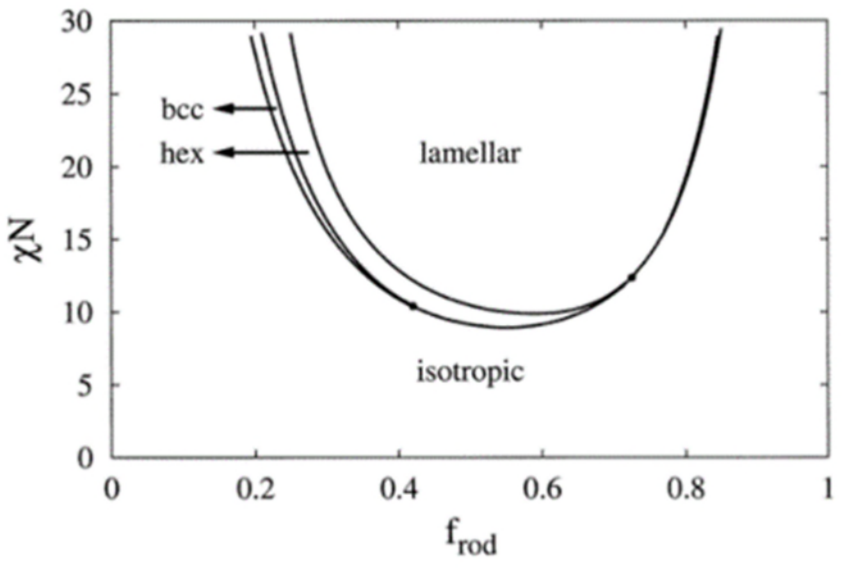

Figure S36. Theoretical phase diagram for a rod-coil block copolymer, taken from ref 3.[3]

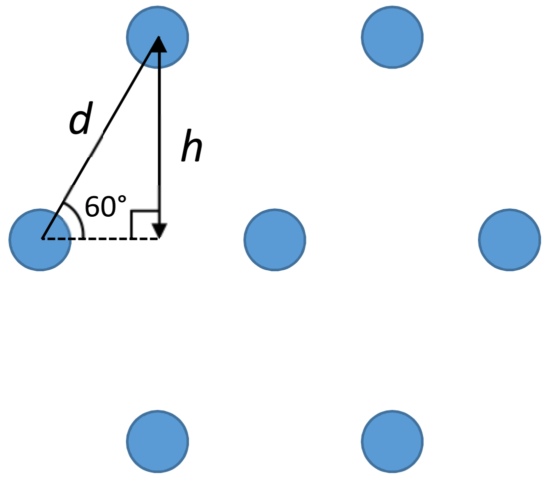

	q^* (Å^{-1})	$h = 2\pi/q^*$ (nm)	$d = h/\sin 60$ (nm)	$d^* = d/2$ (nm)
F_{13}-PAA_9	0.15114	4.16	4.80	2.4
F_{13}-PAA_{11}	0.14079	4.46	5.15	2.6
F_{17}-PAA_{11}	0.12371	5.08	5.87	2.9
F_{17}-PAA_{17}	0.11805	5.32	6.14	3.1
F_{17}-PAA_{23}	0.10691	5.88	6.79	3.4
F_{17}-PAA_{30}	0.09996	6.29	7.26	3.6
F_{21}-PAA_{20}	0.09503	6.61	7.63	3.8
F_{21}-PAA_{24}	0.09381	6.70	7.74	3.9

Figure S37. Calculation of centre-to-centre domain distance for hexagonally packed cylinder morphology. Where *h* is the interplane spacing taken directly from SAXS data, and *d* is the calculated distance between domain centres for hexagonally packed cylinders (domain spacing at full pitch).

References

1 M. Ciampolini and N. Nardi, *Inorg. Chem.*, 1966, **5**, 41–44.

2 E. Hancox, E. Liarou, J. S. Town, G. R. Jones, S. A. Layton, S. Huband, M. J. Greenall, P. D. Topham and D. M. Haddleton, *Polym. Chem.*, 2019, **10**, 6254–6259.

3 M. Reenders and G. Ten Brinke, *Macromolecules*, 2002, **35**, 3266–3280.
